# Conformational dynamics promotes disordered regions from function-dispensable to essential in evolved site-specific DNA recombinases

**DOI:** 10.1016/j.csbj.2022.01.010

**Published:** 2022-01-22

**Authors:** Carla Guillén-Pingarrón, Pedro M. Guillem-Gloria, Anjali Soni, Gloria Ruiz-Gómez, Martina Augsburg, Frank Buchholz, Massimiliano Anselmi, M. Teresa Pisabarro

**Affiliations:** a Structural Bioinformatics, BIOTEC, TU Dresden, Tatzberg 47-51, 01307 Dresden, Germany; b University Carl Gustav Carus and Medical Faculty, UCC, Medical Systems Biology, TU Dresden, Fetscherstrasse 74, Dresden, Germany; c Theoretical Physics and Center for Biophysics, Saarland University, 66123 Saarbrücken, Germany

**Keywords:** Intrinsically disordered protein regions, site-specific DNA recombinase, Evolution, Molecular dynamics, Structural disorder, Thermodynamic stability, AI-based folding

## Abstract

Protein intrinsically disordered regions (IDRs) play pivotal roles in molecular recognition and regulatory processes through structural disorder-to-order transitions. To understand and exploit the distinctive functional implications of IDRs and to unravel the underlying molecular mechanisms, structural disorder-to-function relationships need to be deciphered. The DNA site-specific recombinase system Cre/loxP represents an attractive model to investigate functional molecular mechanisms of IDRs. Cre contains a functionally dispensable disordered N-terminal tail, which becomes indispensable in the evolved Tre/loxLTR recombinase system. The difficulty to experimentally obtain structural information about this tail has so far precluded any mechanistic study on its involvement in DNA recombination. Here, we use *in vitro* and *in silico* evolution data, conformational dynamics, AI-based folding simulations, thermodynamic stability calculations, mutagenesis and DNA recombination assays to investigate how evolution and the dynamic behavior of this IDR may determine distinct functional properties. Our studies suggest that partial conformational order in the N-terminal tail of Tre recombinase and its packing to a conserved hydrophobic surface on the protein provide thermodynamic stability. Based on our results, we propose a link between protein stability and function, offering new plausible atom-detailed mechanistic insights into disorder-function relationships. Our work highlights the potential of N-terminal tails to be exploited for regulation of the activity of Cre-like tyrosine-type SSRs, which merits future investigations and could be of relevance in future rational engineering for their use in biotechnology and genomic medicine.

## Highlights

• New functional regions emerging in evolution of DNA site-specific recombinase tails.
• Transient structural nucleation promotes function-dispensable regions to essential.
• Molecular dynamics reveals conformational diversity and its functional implications.
• Evolved disordered molecular mechanisms of N-term tails for protein stability.
• Structural disorder-based link between protein evolution, stability and function.

## Graphical abstract

**Figure:**
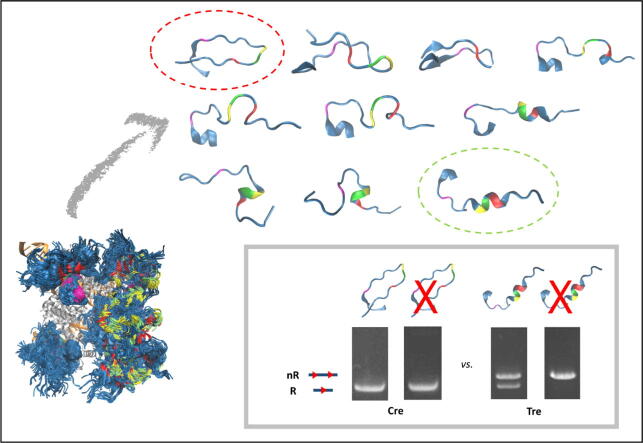

## 1 Introduction

Intrinsically disordered regions (IDRs) challenge the sequence-structure–function paradigm by which the sequence of a protein unequivocally determines a three-dimensional (3D) structure and a function as they can adopt a dynamic ensemble of conformational states that potentially determine diverse functional consequences. IDRs are involved in a variety of cellular functions including allosteric regulation and cooperative binding [1], [2], [3], and they have brought new perspectives in the protein engineering field, since their unique biophysical traits can be utilized to enhance functionalities or to introduce new ones. Consequently, there is an increasing interest in understanding the role of IDRs intrinsic flexibility and structural disorder in molecular mechanisms underlying distinctive functional implications. However, because of their inherent plasticity and lack of unique stable conformation, the characterization of IDRs represents a major challenge in modern structural biology. Although in the last years a great variety of experimental and computational approaches have been applied to characterize IDRs [4], [5], [6], their functional mechanisms remain largely unknown.

Directed molecular evolution has acquired importance in protein engineering, and structural information has helped to improve evolution strategies aimed at the attainment of new customized properties for a diverse set of applications [7]. Including structural information in evolutionary models may drive the comprehension of the evolutionary process, providing mechanistic insights into how proteins can evolve new functions. Accordingly, sequence changes affecting protein structure, yet preserving its stability, have been shown to be key for evolution [8], [9]. Moreover, information obtained from evolutionary studies can also be utilized to enhance our understanding of protein structure adaptability and to help with the disclosure of structure–function relationships, which represent a great value for future engineering endeavors.

In this work, we investigate how evolution and the dynamic properties of IDRs may determine distinct functional properties in two related site-specific DNA recombinase (SSR) systems with the aim of deciphering the molecular mechanisms involved. SSRs hold great promises in the field of biomedicine and biotechnology due to their excellent ability to perform genetic alterations with high efficiency and specificity [10], [11], [12]. Among other methodologies, substrate-linked directed evolution has been used to foster genome engineering by expanding the natural repository of these recombinase systems through the identification of recombinase variants with specificity towards new DNA target sequences [13], [14], [15], [16]. The Cre/loxP SSR system, which is routinely used as a tool for genetic recombination, both *in vitro* and *in vivo* [12], [17], represents an attractive model to investigate functional molecular mechanisms of IDRs as Cre contains a functionally dispensable disordered N-terminal tail [18], which becomes indispensable for the Cre-based substrate-linked evolved Tre/loxLTR recombinase system [19].

**Fig. 1:**
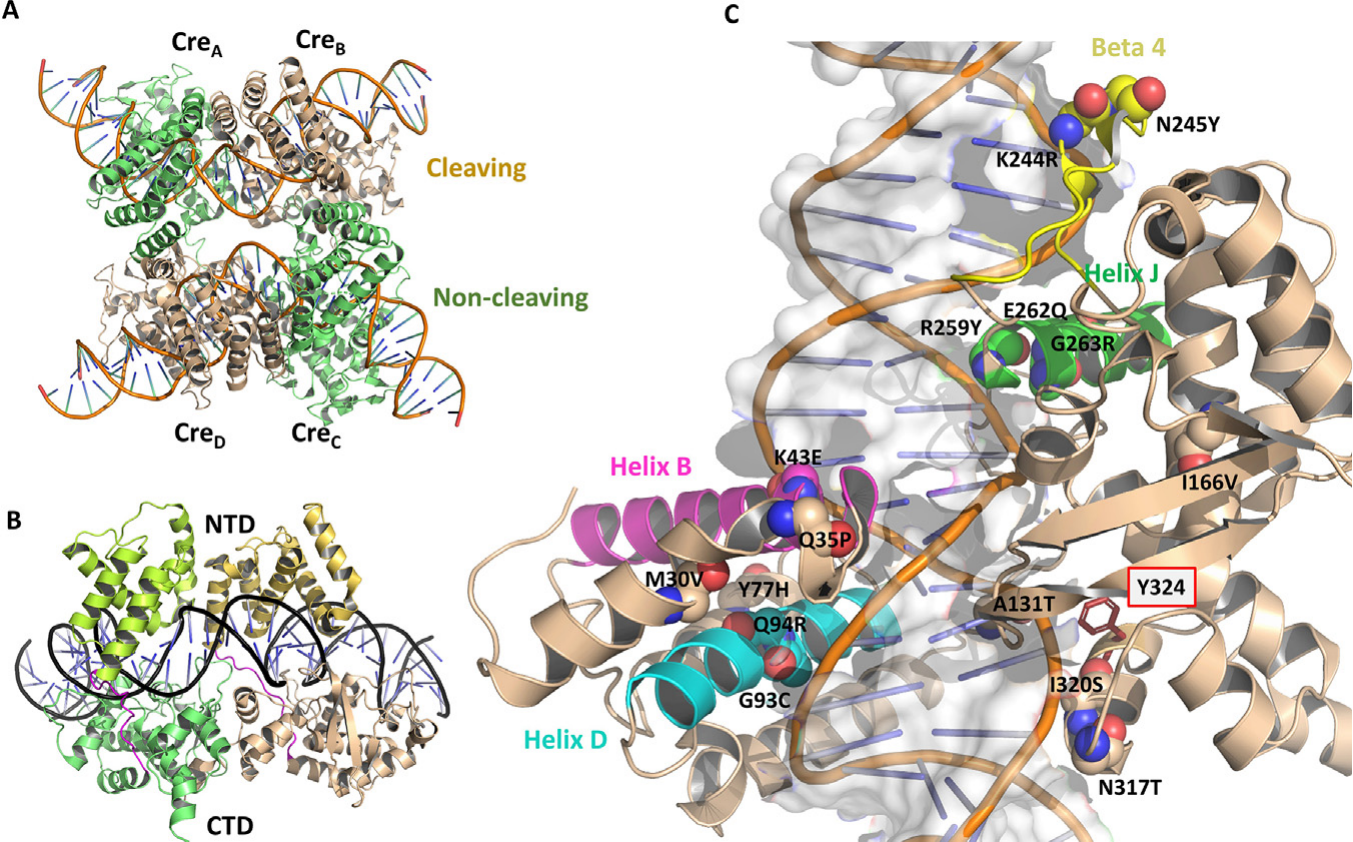
Details of the structure of the tetrameric Cre/loxP synaptic complex (PDB ID 1Q3U). A) Non-cleaving and cleaving Cre monomers (Cre_A_, Cre_C_, and Cre_B_, Cre_D_) are shown in green and brown ribbons, respectively. The DNA backbone is shown as a thin ribbon with the bases displayed in ladder representation. B) View of the N- and C-terminal domains (NTD and CTD) of monomers Cre_A_ and Cre_B_ forming a C-shaped clamp on the loxP site. C) Zoom in on the four mutational hot spot regions observed in the substrate-linked evolution process of Cre-to-Tre towards the new DNA target site loxLTR: helix B (magenta), helix D (cyan), helix J (green), and β-strand 4 (yellow). The 19 amino acids differing between Cre and the evolved recombinase Tre are displayed in CPK spheres, and the respective mutations are labeled. The catalytic residue Y324 is shown in sticks and labeled. Figure generated with PyMOL version 2.4 (Schrödinger LLC; https://pymol.org).

Cre (Causes Recombination) is a 341 amino acids protein of the tyrosine SSR family that specifically targets the 34 base pair (bp) DNA sequence loxP [20]. Cre catalyzes DNA recombination through a multi-step process involving a homotetramer protein complex bound to two loxP sites [21] (Fig. 1A). In the synaptic state, two Cre monomers are in ‘cleaving’ conformation while the other two are in ‘non-cleaving’ conformation. The catalysis proceeds through the formation of a so-called Holliday junction intermediate undergoing isomerization between the cleaving and non-cleaving conformations [22], [23], [24]. Each Cre monomer consists of an N- and a C-terminal domain (NTD, residues 1 to 129, and CTD, residues 132 to 341, respectively), which grab the DNA from either side forming a C-shaped clamp (Fig. 1B) so that the catalytic tyrosine at the CTD can initiate the cleavage and strand exchange reactions.

Tre recombinase was engineered through substrate-linked evolution of Cre to specifically recognize 34-bp within the HIV-1 long terminal repeat regions, loxLTR [25]. The 19 mutations introduced by this evolutive process spread across the protein sequence (Fig. S1); however, the investigation of the mutational frequency obtained in the evolution process led to the identification of four mutational hot spot regions involving helix B, helix D, helix J and β-strand 4 (Fig. 1C) [26]. The analysis of these mutational hot spots in the context of the available crystallographic structures of Cre/loxP [12] and Tre/loxLTR [19], together with the information about other Cre-like naturally occurring and evolved recombinase systems [26], [27], [28], [29], [30] have improved our knowledge on the determinants of molecular recognition in SSRs. Interestingly, in addition to these four mutational hot spots, it was observed that the N-terminal residues of Tre were also frequently mutated along the *in vitro* evolution process, particularly positions 7, 9, 10, and 16 [25], [26]. The N-terminal (Nt) region of tyrosine SSRs is not conserved in nature [31], and in Cre it has been previously reported to be unnecessary for recombination activity in the Cre/loxP system (*i.e.* removal of the N-terminal first twelve (Nt_12_) [32] or twenty (Nt_20_) [18] residues did not affect Cre recombination activity on loxP).

The investigation of the 19 mutations obtained in the evolution of Tre from Cre (Fig. 1C and Fig. S1) by single back mutations resulted in the loss of recombination activity on loxLTR for the majority of residues in the four mutational hot spot regions. Surprisingly, the single back mutations in the Nt tail reduced Tre activity, and particularly the back mutation at position 7 completely abolished Tre recombination on loxLTR [19]. This raised the question of how evolution could exploit the physicochemical properties of this N-terminal tail and thus provide it with an essential functional role in Tre when being unnecessary for activity in its ancestor Cre.

All so-far experimentally available structures of Cre [12] and Tre [19] lack electron density in the Nt tail of the enzyme, which reflects the highly flexible nature of this region. The difficulty to obtain experimentally structural information through X-ray crystallography about this Nt tail has so far precluded any detailed study on its possible involvement in DNA target recognition in Cre-like SSRs. Besides the extensive studies performed on this recombination system by X-ray crystallography [12], several other biophysical experimental and computer-aided studies focusing on DNA topology, kinetics of DNA-loop formation and synapsis geometry have been directed towards acquiring information about the conformational dynamics, stability and topology of reaction intermediates and, thus, the recombination mechanisms of the Cre recombinase and related systems [33], [34], [35], [36], [37]. These studies have provided relevant structural, dynamic and functional mechanistic information on these recombinase systems and a complementary view to the one provided by X-ray crystallography. However, none of these studies has attributed any particular role to the Nt tail of the recombinase.

Here, we make use of *in vitro* and *in silico* evolution data, extensive molecular dynamics (MD) simulations, AI-based folding and thermodynamic stability calculations, mutagenesis and DNA recombination functional assays to *(i)* explore the conformational, dynamic and thermodynamic stability properties of these flexible regions, *(ii)* investigate their distinct functional implications in DNA recombinase activity, and *(iii)* study the possible underlaying molecular mechanisms. Based on the results obtained, we propose a link between protein stability and function and offer new plausible mechanistic insights into disorder-function relationships.

## 2 Results and discussion

### 2.1 Functional characterization of N-terminal IDRs in Cre and Tre.

**Fig. 2:**
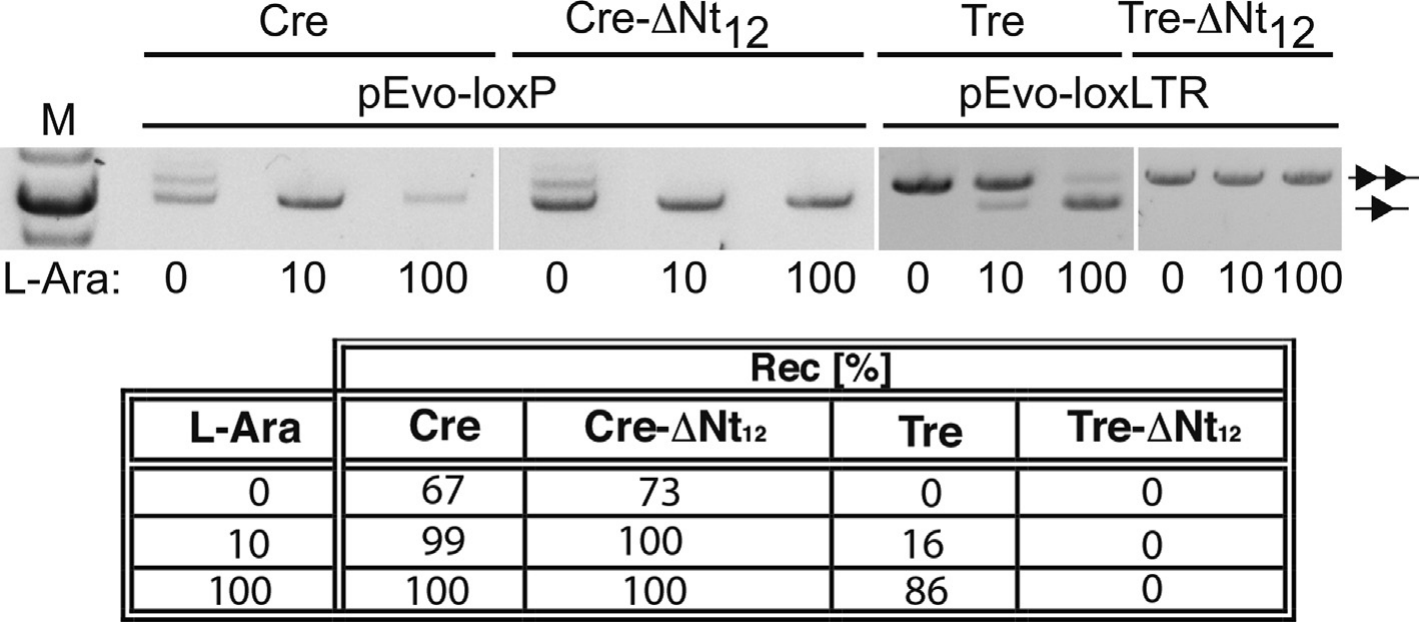
Recombination efficiency of wild-type Cre/loxP and Tre/loxLTR recombinase systems and their respective 12 residue N-terminal deletion mutants (indicated by ΔNt_12_). TOP: Gel showing the employed L-arabinose levels (0, 10 and 100 μg/ml) to induce recombinase expression. The lines with two triangles and one triangle depict the band sizes for non-recombined and recombined plasmids, respectively. M = marker (1 kb ladder). BOTTOM: Quantification of recombination efficiencies in *E. coli*. Recombination (in %) was calculated from measuring gel band intensities for indicated recombinases.

To compare the functional relevance of the N-terminal tail of Cre and Tre (Cre-Nt and Tre-Nt, respectively), we generated recombinase versions in which the 12 first residues were deleted by site-directed mutagenesis (Cre-ΔNt_12_ and Tre-ΔNt_12_) and tested their recombination activity on loxP and loxLTR in a plasmid-based assay, respectively (Fig. S2). As previously reported [32], the Nt_12_ deletion had no effect on the recombination activity of Cre on loxP (Fig. 2). In contrast, the Nt_12_ deletion on Tre completely abolished recombination on loxLTR, in line with the already reported inactivity of single back-to-Cre mutations in the Tre N-terminus [19]. Likewise, the Nt_12_ deletion in a Tre mutant (Tre_P35Q_), previously reported to have increased activity compared to Tre [19], and in the evolved Brec1 recombinase [26] (Fig. S1) also showed a complete loss of activity of these recombinases on their respective DNA target sites (*i.e.* loxLTR and loxBTR, respectively) at all L-arabinose concentrations tested (Fig. S3).

These results establish the important functional role of the first 12 residues of the evolved Tre recombinase and suggest that the substrate-linked *in vitro* evolution process might have assigned new properties granting such a functional role, which is non-existent for the Nt tail of wild-type Cre.

These findings sparked our interest in studying in a detailed and comparative manner the conformational and dynamic properties of these IDRs to investigate the possible molecular mechanisms responsible for the functional differences between Tre and Cre, as it might offer important clues and guidance for the future rational development of new designer SSRs.

### 2.2 Conformational characterization of N-terminal IDRs in Cre and Tre.

The Nt region of Cre and Tre recombinases (Fig. S1) exhibits high intrinsic flexibility. In the so-far experimentally available structures of Cre [12] and Tre [19] in the Protein Data Bank (PDB; https://www.rcsb.org/) [38], only some entries have coordinates for residues 11 to 20, and not in all the monomers of the t-etrameric complex. The lack of any fully resolved Nt tail in the PDB prompted us to investigate the conformational properties of this IDR following a sequence homology, secondary structure, and AI-based folding prediction as well as an extensive MD-based analysis.

#### 2.2.1 Conformational sequence-, secondary structure- and AI folding- based analysis.

In a first attempt to investigate the intrinsic conformational propensities of the first 20 residues of Cre and Tre (Cre-Nt_20_ and Tre-Nt_20_, respectively) (Fig. S1), we performed a Blast [39] protein sequence comparative homology search with all proteins of known structure available in PDB (see Materials and Methods for details). Among the obtained top hits, the sequence homology search with Cre-Nt_20_ identified a 100% sequence identity with residues 3 to 10 in PDB entry 4FBK, which were adopting an extended conformation in the context of a buried β-sheet (Fig. S4 A). The search with Tre-Nt_20_ identified an α-helical fragment immersed in a helical bundle exhibiting 90% sequence identity with residues 6 to 14 in PDB entry 5WSG (Fig. S4 B). Similar conformational properties for the two Nt_20_ regions were obtained by secondary structure predictions performed with CFSSP [40] and PSIPRED [41] (see Materials and Methods for details) (Fig. S4 C-D).

Interestingly, folding predictions performed for the Nt region of Cre and Tre with the recently released AI-based AlphaFold methodology [42], [43] (see Materials and Methods for details) showed a structurally organized helical Nt tail packing against helix A for Tre versus a non-structurally defined Nt tail for Cre, which was not establishing any interaction with helix A (Fig. S4 E-F).

The preliminary conformational predictions obtained from these 3 different methods should be interpreted carefully (*i.e.* in a different protein context a given sequence may behave differently). Nevertheless, they all pointed towards a certain, at least partial, intrinsic α-helical propensity for Tre Nt tail whereas disordered for Cre.

#### 2.2.2 Conformational ST MD-based analysis.

**Fig. 3:**
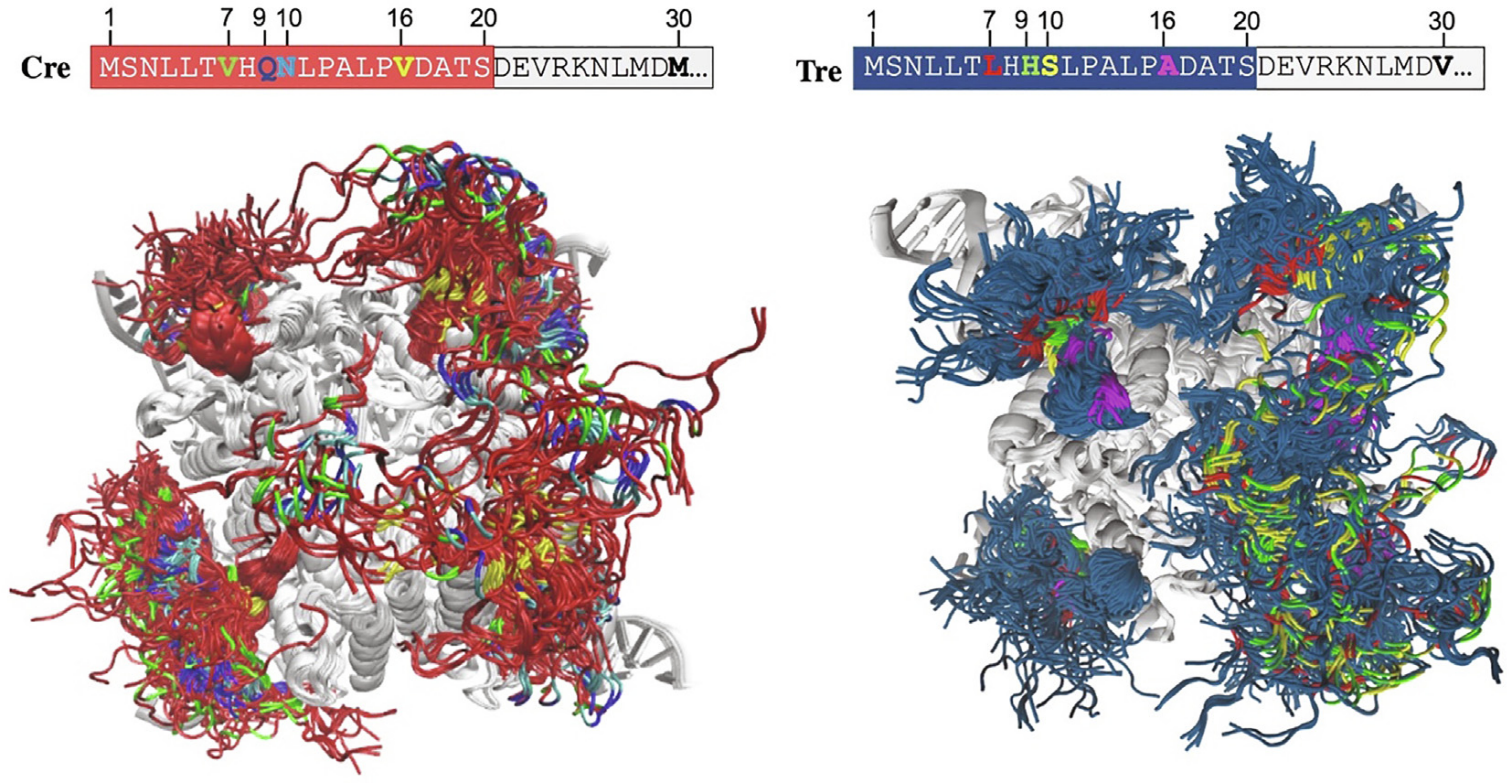
TOP: Amino acid sequence of the N-terminal tail of Cre (left) and Tre (right). Relevant amino acid positions are labeled. Differing residues are highlighted in bold and in a color code that will be used for all molecular models (*vide infra*). BOTTOM: Structural ensemble obtained from the ST MD simulations for Cre-Nt_20_/loxP (left) and Tre-Nt_20_/loxLTR (right). The backbone of residues 1 to 20 is displayed in red for Cre (left) and in blue for Tre (right), and the rest of the protein and the DNA are shown in gray. For differing amino acids in the Nt_20_, the same color code is used as in the sequence in the top panel. Figure generated with VMD [47]. (For interpretation of the references to colour in this figure legend, the reader is referred to the web version of this article.)

*Molecular modeling of Nt_20_ and ST MD simulations.* We conducted extensive MD simulations to investigate in more detail the conformational properties of the first twenty amino acids in Cre and Tre (Fig. 3 and Fig. S1). For this purpose, we used the available crystal structures of Cre and Tre in complex with their native DNA target sites (PDB ID 1Q3U [21] and 5U91 [19], respectively). In each recombinase/DNA complex structure,residues 1-20 (Nt_20_) were modeled in random extended conformation for each of the four protein monomers (see Materials and Methods for details).

MD simulations were carried out for each protein/DNA complex for a total of 1.3 μs using GROMACS [44], [45] and a simulated tempering (ST) MD approach [46] to generate structural ensembles of the unrestrained Nt_20_ region in both enzymes, while the rest of the protein and DNA remained restrained during the simulation (for details, see Materials and Methods). The structural ensembles obtained from these ST MD simulations for Cre-Nt_20_ and Tre-Nt_20_ are shown in Fig. 3. Visual inspection indicated great flexibility and conformational versatility of the N-terminal tails.

**Fig. 4:**
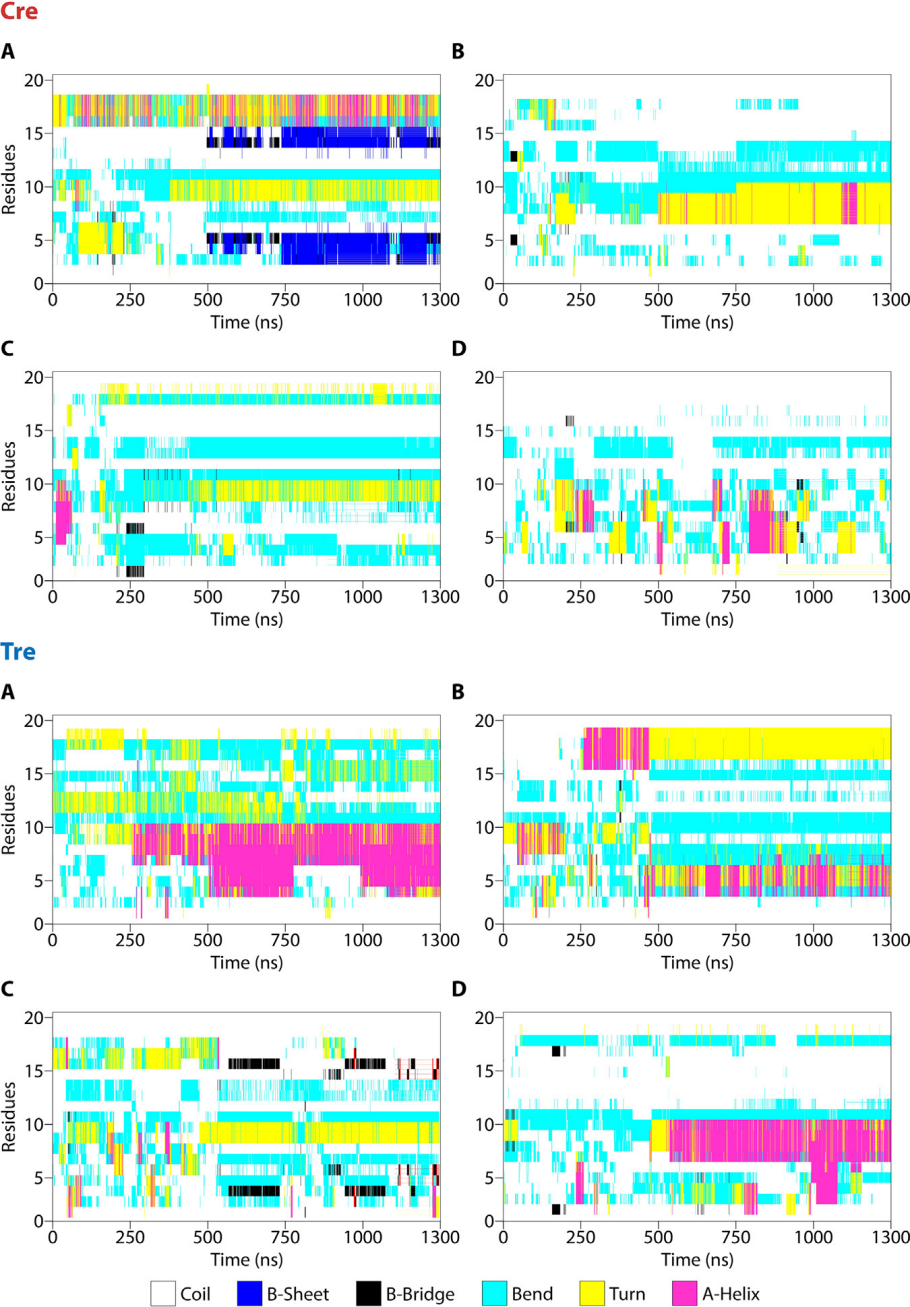
DSSP-based [48] secondary structure analysis of Nt_20_ in each of the four monomers of Cre (TOP: Cre_A_ (A), Cre_B_ (B), Cre_C_ (C), Cre_D_ (D)) and of Tre (BOTTOM: Tre_A_ (A), Tre_B_ (B), Tre_C_ (C), Tre_D_ (D)). Data analyzed was obtained from the 1.3 μs ST MD simulation with GROMACS [44], [45] at 300 K.

*ST MD-based secondary structure analysis.* The secondary structure configuration of the conformations obtained for Cre-Nt_20_ and Tre-Nt_20_ along their corresponding ST MD trajectory at 300 K was analyzed with DSSP [48]. The results obtained for each of the four Cre and Tre monomers (Cre_A_, Cre_B_, Cre_C_, Cre_D_, and Tre_A_, Tre_B_, Tre_C_, Tre_D_, respectively) are shown in Fig. 4, which reflect secondary structure configurational differences among the monomers.

In Cre-Nt_20_, although few residues showed some tendency to form a stable turn, there was, in general, no strong secondary structure formation observed throughout the MD simulation, signifying the highly flexible and unstructured nature of the Nt tail of Cre. For Cre_A_ (Fig. 4 A, TOP), a few Nt residues showed a propensity to form an extended β-strand structure after 500 ns. However, such a propensity was not observed in any of the other protein monomers (*i.e.* Cre_B_, Cre_C_ and Cre_D_; Fig. 4 B-D, TOP). The results obtained for Tre-Nt_20_ showed a different conformational behavior. Monomers Tre_A_ and Tre_D_ exhibited a clear formation of α-helical structures after 500 ns, which remained folded through the complete simulation time (Fig. 4 A,D, BOTTOM). Monomer Tre_B_ also showed a tendency to form α-helix during the simulation although with less stability (Fig. 4 B, BOTTOM). Overall, our analysis indicated that the first twenty residues in Cre have the tendency to remain unstructured, and that there is a certain extended β-strand conformation being nucleated but observed only in one monomer after 500 ns. In Tre, however, our analysis revealed a clear tendency of its Nt to nucleate α-helical conformations and, in some instances, even adopt a stable α-helix fold.

These results are in line with our preliminary sequence-, secondary structure- and AI-based folding predictions (section 2.2.1). Hence, the physicochemical property changes introduced during the Cre-to-Tre evolution process in positions 7, 9, 10 and 16 (Fig. 3 and Fig. S1) should be investigated in the context of their configurational variability in order to elucidate their possible role in emerging the function of the Nt in Tre.

**Fig. 5:**
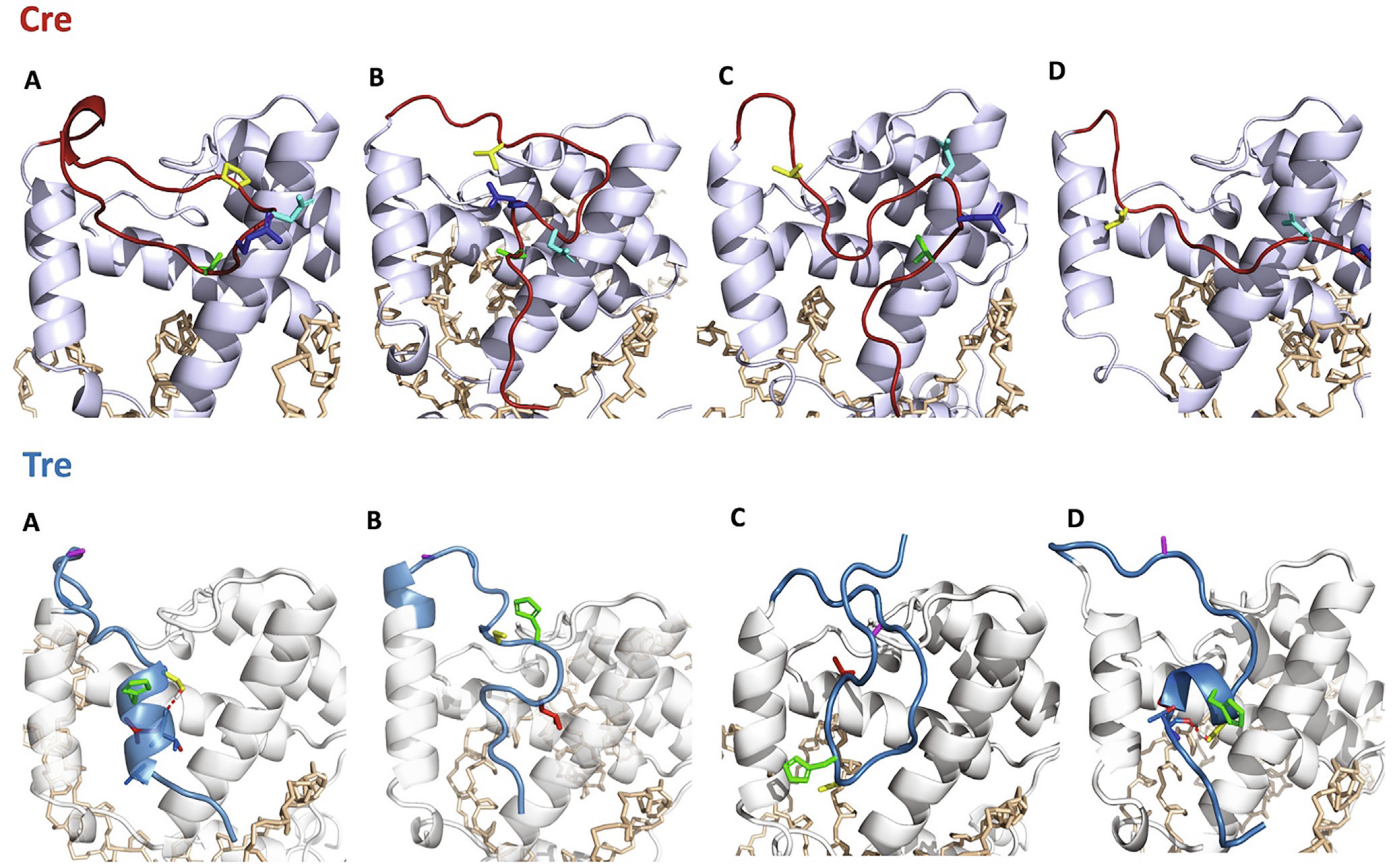
Representative structures for each monomer of the topmost populated Nt_20_ clusters obtained from the conformational ensemble extracted from the ST MD trajectory at 300 K. TOP: Representative Cre-Nt_20_ structure for monomers Cre_A_ (A), Cre_B_ (B), Cre_C_ (C) and Cre_D_ (D). BOTTOM: Representative Tre-Nt_20_ structure for monomers Tre_A_ (A), Tre_B_ (B), Tre_C_ (C) and Tre_D_ (D). The Nt_20_ is shown in red and blue, and the rest of the protein in a gray and white ribbon for Cre and Tre, respectively. The DNA backbone is shown in beige sticks. The side chains of the amino acids at positions 7, 9, 10 and 16 are shown in sticks in the same color code used in Fig. 3. Red dashed lines indicate hydrogen bonds. Figure generated with PyMOL version 2.4 (Schrödinger LLC; https://pymol.org/). (For interpretation of the references to colour in this figure legend, the reader is referred to the web version of this article.)

*ST MD conformational clustering analysis and selection of representative configurations.* Clustering analysis was performed to investigate in detail the plethora of conformations exhibited by Cre-Nt_20_ and Tre-Nt_20_ along their respective complete ST MD trajectory. The analysis was performed for each of the four monomers in each recombinase/DNA system (see Materials and Methods). Representative Nt_20_ structures of the topmost populated configurational clusters were selected for each of the four monomers of Cre and Tre (Fig. 5). Their detailed analysis revealed no structural order propensity in the N-terminal tail of Cre (Cre-Nt), in contrast to Tre for which a tendency of its N-terminal tail (Tre-Nt) to form stable short α-helices was clearly observed.

In Cre, the appearance of the most populated clusters was 42%, 47%, 60% and 1 % for Cre_A_, Cre_B_, Cre_C_, and Cre_D_, respectively. In Tre, the most populated clusters exhibited a frequency of 22%, 62%, 33% and 31 % for Tre_A_, Tre_B_, Tre_C_, and Tre_D_, respectively. As observed from the DSSP secondary structure analysis of the ST MD (*vide supra*, Fig. 4), the clustering analysis evidenced an extended or unfolded Nt_20_ tail for Cre (Fig. 5 A-D, TOP), whereas in the case of Tre-Nt_20_ the formation of short α-helices was observed in monomers Tre_A_ and Tre_D_, while in the other two monomers the Nt_20_ region remained in random coil conformation (Fig. 5 A-D, BOTTOM).

In conclusion, the conformational characterization of the Nt in Cre and Tre performed with different approaches suggested remarkable conformational differences. The high-disordered nature of the configurations observed for Cre-Nt_20_ and the dynamic disordered-ordered structural versatility obtained for Tre-Nt_20_ would explain the lack of an even diffraction pattern and electron density in their respective crystal structures, which would be necessary to resolve the structure of this Nt tail. Furthermore, our configuration analysis evidences conformational preferences for Tre-Nt_20_ that might have relevant functional implications. It is worth noticing that only a part of the Nt_20_ region in Tre is predicted as folded. The plethora of conformations observed for each of the four monomers could resemble different stages of the folding behavior of this IDR. As such, residue substitutions introduced by the evolutive process might have conferred the needed features to adopt a particular 3D disposition responsible for conferring the essential functionality observed. With this in mind, we performed further analysis of the representative configurations. A detailed structure–function analysis of the mutations introduced in Tre-Nt_20_ during the evolution process and a comparative investigation of their differences with respect to wild-type Cre-Nt_20_ (*i.e.* V7L, Q9H, N10S and V16A; Fig. 3 and Fig. S1) could help to establish the molecular mechanisms behind the functional relevance of this IDR.

### 2.3 Evolution-disorder–order-function relationships rationale.

**Fig. 6:**
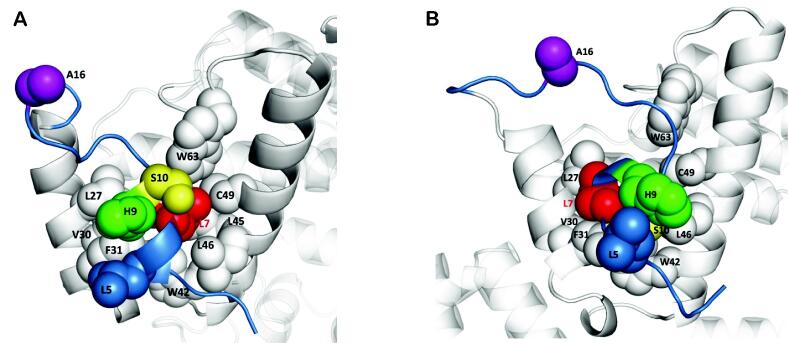
Details of the packing of the folded Nt_20_ region in Tre_A_ (A) and Tre_D_ (B) with the rest of the protein. The structures correspond to representatives of the conformational ensemble extracted from the ST MD trajectory. Relevant amino acid side chains are shown in CPK representation and labeled. Nt residues are colored following the same code as in Fig. 3. Figure generated with PyMOL 2.5.0 (Schrödinger LLC; https://pymol.org/). (For interpretation of the references to colour in this figure legend, the reader is referred to the web version of this article.)

Visual inspection of positions 7, 9, 10 and 16 (*i.e.* differing amino acids in the Nt; Fig. 3 and Fig. S1) in the representative Cre-Nt_20_ structures obtained from the ST MD simulation did not show any clear interaction pattern with the rest of the protein. In contrast, in the representative structures of Tre-Nt_20_, it was observed that the formation of short α-helices at Nt promoted its packing against a “V-shaped” hydrophobic conserved surface between helices A and B (Fig. S1) forming a mini-hydrophobic cluster including residues L5, L7, L27, V30, F31, W42, L45, L46, and W63, which was noticeable and well-defined in monomers Tre_A_ and Tre_D_ (Fig. 6 A and 6 B, respectively).

The structural ordering of the Nt tail and its packing against the protein forming a mini-hydrophobic core could eventually stabilize the protein’s NTD. It is worth noting that residues L7 and V30 in Tre are two of the 19 mutations that appeared during the Cre-to-Tre evolution process (*i.e.* V7L and M30V; Fig. S1). Interestingly, back mutating (*i.e.* Tre-to-Cre) residue 7 to valine (*i.e.* L7V) has been shown to completely abolish Tre activity on loxLTR, whereas back mutation of residue 30 to methionine (*i.e.* V30M) increases Tre’s activity [19].

Regarding the other substitutions at the N-terminus by the Cre-to-Tre evolution (*i.e.* 9, 10, 16; Fig. 3 and Fig. S1) that showed a decrease of activity of Tre on LoxLTR upon single back-to-Cre mutations [19], our models indicated that serine at position 10 could also play a role in stabilizing the short α-helix in Tre-Nt_20_, as its side chain was observed forming an intra-helical hydrogen bond with the main chain of Thr6 in monomers Tre_A_ and Tre_D_. Alanine at position 16 might enhance flexibility in the loop connecting the Nt tail and helix A. Further analysis of the Tre_A_ and Tre_D_ structures showed that histidine at position 9 is solvent-exposed; therefore, no difference could be inferred from glutamine at this position in Cre (Fig. 6).

Our studies suggest that the mutations introduced by the Cre-to-Tre substrate-linked *in vitro* evolution helped to turn a highly disordered and functionally dispensable framework at the N-terminus in Cre into a conformationally dynamic but certainly defined and functionally essential architecture in Tre, and it could be hypothesized that a plausible mechanism behind such change could be the stabilization of Tre NTD structure through the formation of a hydrophobic mini-core between the V-shaped site formed by helices A and B and the N-terminus. This hypothesis would diverge from what was initially thought after the substrate-linked evolution process about a presumed involvement of these Nt residues in forming direct contacts with the new DNA target site. In fact, the scenario observed from our ST MD simulations is different, as neither in Cre nor in Tre the N-terminus interacts with the DNA. Instead, in Cre the side chain of M30 is packed intimately with the neighboring hydrophobic residues, while the Nt tail appears highly disordered, with the side chain of V7 solvent-exposed. On the contrary, in Tre the N-terminus is packed against the NTD, with L7 participating in the hydrophobic mini-core.

A change of amino acid sequence allowing a transient structural nucleation combined with a new 3D disposition of the N-terminus allowing interaction with other parts of the protein could be a plausible mechanism to provide the IDR Tre-Nt_20_ with a new functionally essential role. An interplay between substitutions at positions 7 and 30 and the rest of the protein might have been promoted for guaranteeing stability and with that even evolvability, since enzyme stabilization has been previously reported as an important factor for evolvability [49], [50], [51]. In order to further substantiate this evolution-disorder–order-function relationships hypothesis, further detailed investigations were carried out. First of all, by analyzing the behavior of the full protein/DNA complex through classical MD simulations and, secondly, by carrying out *in silico* evolution studies in the Nt region and examining the energetics obtained.

### 2.4 Classical MD simulations of the full protein-DNA complexes.

We performed extensive classical MD simulations to investigate the conformational dynamics properties of the Nt tail in the context of the unrestrained complete recombinase (*i.e.* residues 1–341)/DNA complex. For this, we generated the complete structures of the Cre_1-341_ and Tre_1-341_ recombinases by merging residues 1 to 20 from the corresponding Nt_20_ representative structures to each of the protein monomers in the respective crystallographic structure in complex with the corresponding DNA target site (see Materials and Methods for details). The resulting full-protein/DNA tetrameric complexes were simulated for 0.5 μs each using a classical MD approach in which DNA and protein atoms were kept unrestrained (see Materials and Methods for details). An exception of positional restraints was applied to the C_α_ atoms of residues Arg32 and Glu69, which form a salt-bridge interaction that has been reported to be important for stabilization of the non-cleaving/cleaving monomers interface [19] (Fig. S5). Indeed, without these restraints, a certain dislocation of the interfacial protein residues was observed with a tendency of the protein monomers to go apart with the incremental simulation time. Visual inspection of the trajectories obtained from these classical MD simulations confirmed the great flexibility and conformational versatility of the Nt tail (*vide infra*).

**Fig. 7:**
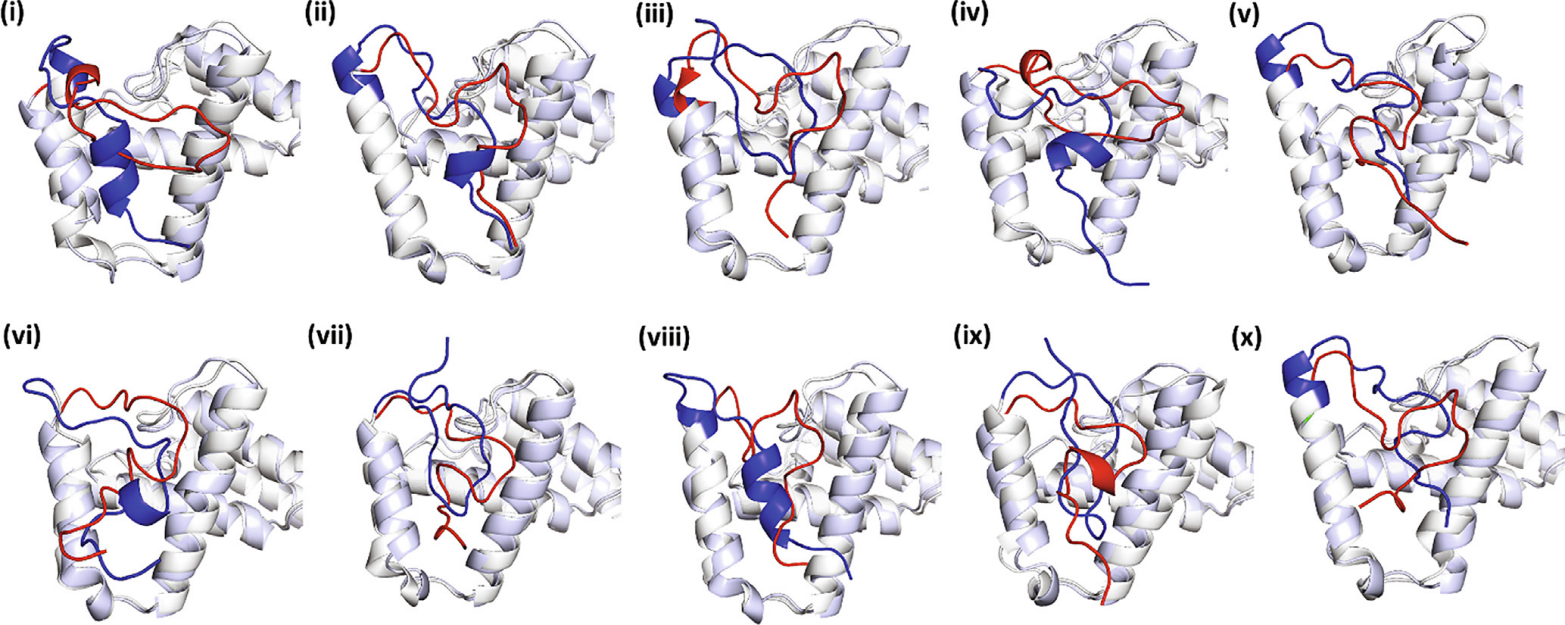
Structural superposition of the NTD (residues 1–129) of the representative structures of the top 10 most populated clusters ((i) to (x), in order of extent of population being *i* the highest) obtained from the 0.5 μs classical MD simulation of the full Cre_1-341_/loxP and Tre_1-341_/loxLTR complexes. Gray and white ribbons represent Cre and Tre, respectively. Nt_20_ is shown in red for Cre and blue for Tre. Figure generated with PyMOL version 2.4 (Schrödinger LLC; https://pymol.org/).

*Conformational clustering analysis and selection of representative configurations.* Clustering analysis was performed in order to investigate in detail the plethora of configurations exhibited by Cre and Tre through the 0.5 μs MD trajectory and to evaluate the preferred conformations for their respective Nt tails. In order to determine the most frequent conformations of the Nt_20_ collectively from all protein monomers, cumulative trajectories were generated containing the corresponding MD trajectories of the four protein monomers of each recombinase/DNA complex (see Materials and Methods). Inspection of the representative structures of the top 10 most populated clusters appearing during the 0.5 μs simulations showed a similar dynamic configuration of the Nt tail to the obtained from the Nt_20_ ST MD simulation (see section 2.2.2). In Cre, the Nt tail was observed completely disordered, whereas in Tre the Nt region exhibited in most cases partially folded structures and certain packing against helices A and B (Fig. 7).

Furthermore, these simulations further confirm that the residues in the Nt tails do not interact with the DNA (*i.e.* no populated states were observed at contact distance), neither in the Cre/loxP nor in the evolved Tre/loxLTR recombinase system, ruling out the idea of the involvement of the Nt tail in gaining specificity towards the new DNA sequence. These findings, together with the observation that apparently the Nt tail does not affect the catalytic site, corroborate the hypothesis that the evolutive amino acid replacements at these IDRs might have a different role.

Might therefore the ordering of the Nt tail in Tre and its consequent packing against the NTD of the recombinase’s structure represent a stabilizing energetic compensation for the accumulation, during the substrate-linked evolution process, of mutations promoting new function but which may be compromising protein stability? There are several studies on how mutations affect protein stability and how stability affects protein evolution (reviewed in [52]). New-function mutations can undermine the thermodynamic and kinetic stability of a protein, so that the requirement for stability and avoidance of misinteractions become the major constraint on protein evolvability. Therefore, the trade-off between the gain of a new function and the protein stability plays a key role in evolutionary dynamics [52], [53], [54], [55]. In an evolutive process, together with mutations promoting function, compensatory mutations (*i.e.* changes in sequence with the ability to suppress the deleterious effects of other mutations and therefore to restore stability) are often observed as a counterpart, thus enhancing the evolution pathways so as to be promoted through evolution. For instance, stabilized variants of P450 and TEM-1 were described to show higher evolvability through their ability to accommodate a larger variety of new-function mutations without loss of enzyme levels [50]. Compensatory mutations have been observed in natural and also *in vitro* evolution [52]. The number of sequences encoding for a given structure generally decreases with native stability. Thus, protein sequences were constrained in evolutionary paths in order to avoid low stability [9], [56]. Furthermore, amino acid substitutions are constrained differently depending on their local environment (*i.e.* secondary structure, solvent accessibility, packing, H-bonding; reviewed in [57]). Therefore, IDRs represent a wide plethora of possibilities in this aspect. Evolutionary simulations investigating the consequences of marginal thermostability in proteins have indicated that the natural tendency of proteins toward marginal stability, and the range of stabilities occurring during evolution, may have a significant effect on the evolutionary process [9], [58].

Based on our MD-based analysis, we propose a plausible molecular mechanism behind the dispensability of Nt_20_ in Cre and its essential functional relevance in Tre through dynamic nucleation of structural order and the disposition of particular functionalities in 3D space, providing stability to the protein. In the studied SSRs systems, with the stability of the evolving enzyme possibly being compromised, the intrinsically disordered and functionally dispensable N-terminal tail might perhaps become a stability enhancer toolkit for the evolution process as it might potentially accommodate changes during evolution more easily and without affecting the new function. On the other hand, a large excess of stability might reduce evolvability, for instance by rigidifying the protein and restricting alternative conformations that mediate the new function (*i.e.* a significant fraction of mutations in the native protein might lead to increased stabilities). Therefore, a partially disorganized tail and a conjugation of order–disorder transitions appear as quite convenient machinery for this purpose. The amino acid substitutions in the Nt tail and the observed interplay with other residues of the NTD could potentially preserve the overall protein stability and, with that, safeguard the effect of new-function mutations and possibly promote evolvability [50]. We therefore decided to investigate possible relationships between the functionalities introduced at the Nt tail by the Cre-to-Tre *in vitro* evolution process and their 3D disposition.

### 2.5 *In silico* evolution of Nt_20_.

We set up an *in silico* evolution framework making use of high-performance computing (HPC) to investigate from an evolutive and energetic perspective a possible correlation between the functionalities introduced at the Nt tail by the Cre-to-Tre *in vitro* evolution process (Fig. S1) and their 3D disposition. Each monomer of the representative Tre structure obtained from ST-MD was mutated in positions 7, 9, 10, 16 and 30 (*i.e.* every position mutated at Nt in the Cre-to-Tre evolution) to each of the 20 canonical amino acids, which resulted in a total of 3.2 million mutant sequences and 12.8 million decoy 3D structures. The Rosetta relax application [59] was used to obtain per-residue energies from positions 1 to 33 (see Materials and Methods for details). The obtained energy landscape for the Nt tail was investigated for each monomer. A histogram using an energy-bucket size of 10 ΔG REU (Rosetta Energy Units) was built for each monomer in order to inspect the overall energy distribution and the frequency of favorable mutations (see Fig. S6). The decoys corresponding to the sequence of Tre were found to score better than the decoys with the sequence of Cre in each of the four monomers. Therefore, as selection criteria, the energy score of Cre was used as a cutoff to extract a subset of “best decoys” for each monomer. The decoy population of each monomer was filtered using their energy value by discarding decoys with a score worse than Cre (see Table S1).

**Fig. 8:**
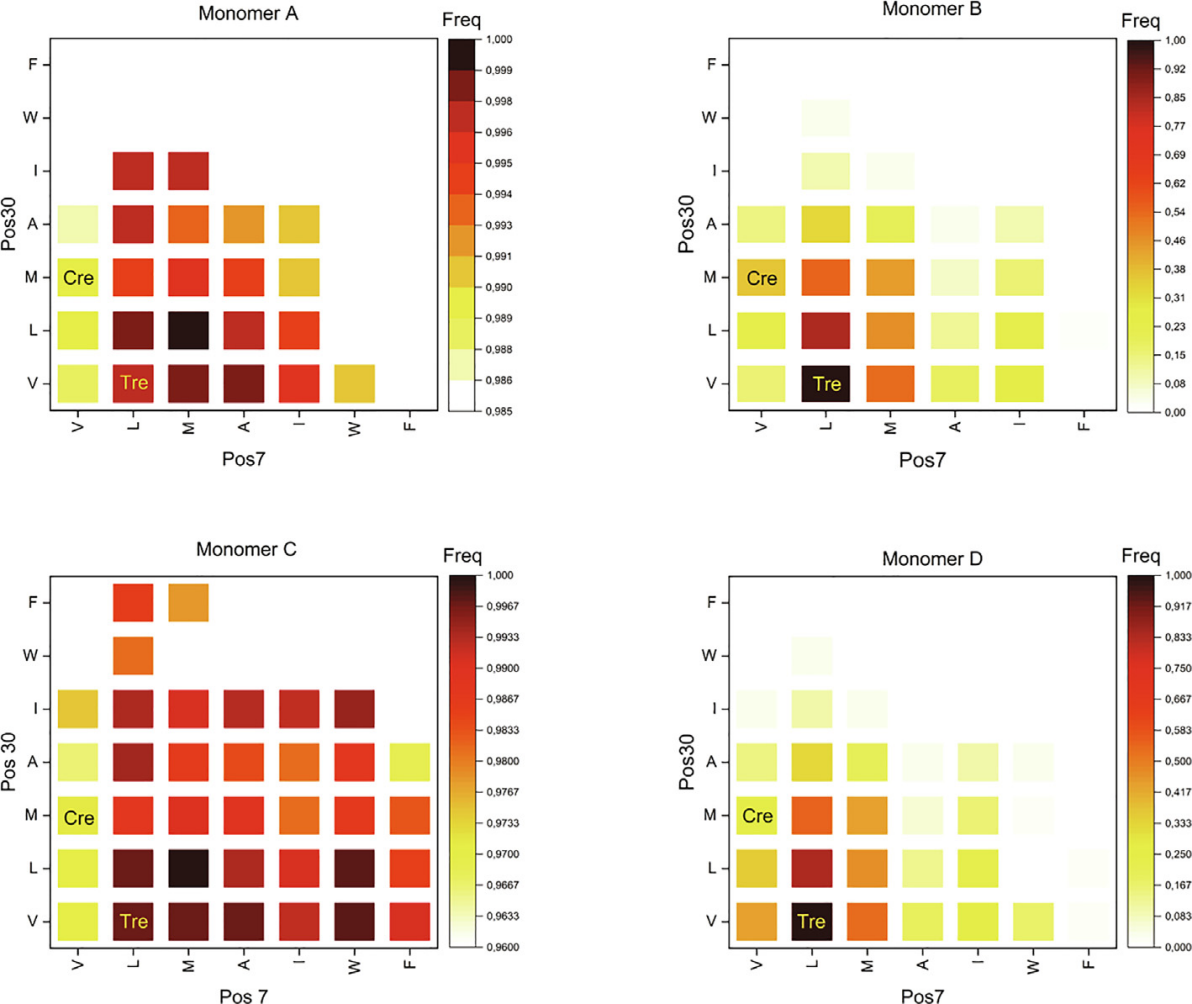
Frequency heatmap of hydrophobic residue combinations in positions 7 and 30 for monomers Tre_A_, Tre_B_, Tre_C_, and Tre_D_. The white/light yellow to red/dark brown color gradient represents low to high frequency of occurrence. The residue combinations corresponding to Cre and Tre are labeled. (For interpretation of the references to colour in this figure legend, the reader is referred to the web version of this article.)

A frequency heatmap was built for each monomer using its corresponding subset of “best decoys” and reporting occurrence frequency and corresponding energy for hydrophobic-residue combinations in positions 7 and 30 (Fig. 8). The broader frequency range observed for monomer A, B and C in contrast to the narrower frequency range observed for monomer D could be explained by the varying number of “best decoys” on each subset in relation to the distance between positions 7 and 30. It can be observed that for monomers Tre_A_ and Tre_D_ (those adopting a folded Nt in the ST-MD simulations) favorable residue permutations occur more frequently around the lower left area of the heatmap, indicating that residue pairs containing Val, Leu and Met tend to increase in frequency in decoys with the best energies. Likewise, the bulky side chains of Trp and Phe seem to occur infrequently due to obvious steric clashes in the structured monomers (A and D). In contrast, the non-structured monomers (B and C) are more permissive with more permutations seen equally frequent. Here, additional permutations in positions 7 and 30 with bulky aliphatic or aromatics may occur without affecting the total score. On the contrary, a clear selective pattern of favorable substitutions for hydrophobic packing is observed for positions 7 and 30 in monomers A and D. The predicted favorable combinations could be endorsed when considering constant the accessible conformational space of the N-terminal tail, which will not be the case when the Nt tail is free in solution, and considering the same folding tendency of the amino acids being substituted. In the configuration of monomer D, our results indicate that favorable energy conformations occur most frequently with L7-V30 combinations, which coincides with the wild-type sequence of Tre. In the configuration of monomer A, this L7-V30 combination also shows high frequency among other hydrophobic non-bulky combinations and their permutations at these two positions.

It is interesting to note that the combination M7-L30 appears with the highest frequency for the configuration in monomer A and C (Fig. 8 and Table S3). Noteworthy, this residue pair is a permutation of the Tre mutant having L7-M30 and reported to increase Tre activity in Tre-to-Cre back mutational experiments [19]. The obtained results are in line with our structure-based hypothesis and the cross-talk between residues of the Nt tail and the NTD, which could potentially enhance the recombinase’s stability.

### 2.6 Thermodynamic stabilities

In order to investigate the contribution of the Nt_20_ tail to the overall stability, we carried out thermodynamic stability calculations with Rosetta [60], [61], [62]. Calculations were performed with iteration sets previously shown to yield the most accurate predictions in benchmarking of stability-prediction algorithms [63].

**Fig. 9:**
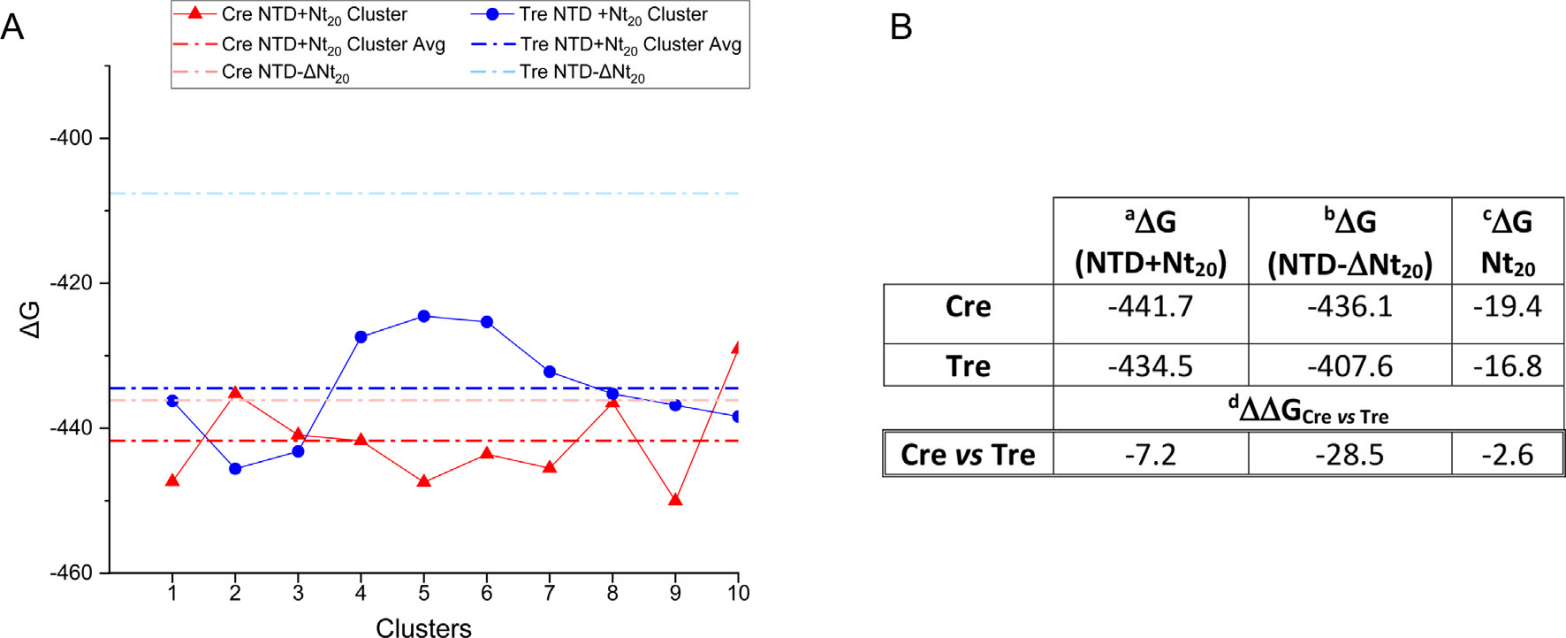
(A) Thermodynamic stabilities obtained with Rosetta for the NTD of Cre (red) and Tre (blue) with and without the Nt_20_ region. The light dashed lines represent the average energy of the NTD of all four monomers without Nt_20_ (Cre NTD-ΔNt_20_ and Tre NTD-ΔNt_20_ in red and blue, respectively) obtained with the respective crystal structure (PDB ID 1Q3U and 5U91, respectively). The dark dashed lines represent the average energy of the NTD of all four monomers with Nt_20_ (in red Cre NTD+Nt_20_ and in blue Tre NTD+Nt_20_) averaged from the 10 top clusters obtained from MD simulations. The scatter plots represent the average energy of the NTD of the four monomers with Nt_20_ per cluster (in red and blue for Cre and Tre, respectively). Energy values are given in Rosetta Energy Units (REU). (B) Contribution of the Nt_20_ tail to the overall stability. ^a^Calculated energies for the NTD with Nt_20_ (NTD+Nt_20_). ^b^Calculated energies for the NTD without Nt_20_ (NTD-ΔNt_20_). ^c^Calculated energies of Nt_20_ alone. ^d^Differences in energy (ΔΔG) when comparing Cre versus Tre.

The thermodynamic stabilities obtained for the NTD with (NTD+Nt_20_) and without Nt_20_ (NTD-ΔNt_20_) as well as for the Nt_20_ alone and averaged for all four monomers (see Materials and Methods for details) are shown in Fig. 9. The Cre and Tre NTD structures containing the Nt_20_ tail showed a moderate difference in their overall thermodynamic stability (*i.e.* ΔΔG = −7.2 REU). On the other hand, the values obtained for the NTD without the Nt_20_ tail showed a remarkable difference between Cre and Tre (ΔΔ*G* = −28.5 REU). We assume that this large difference is due to the absence of Nt_20_ packing with the rest of the protein, as the values obtained for the Nt_20_ alone in Cre and Tre are similar (*i.e.* ΔΔG = −2.6 REU). Based on these observations, we conclude that the contribution of the Nt_20_ tail to the overall stability of Tre is due to both, its presence and folding as well as its packing against the hydrophobic V-shaped conserved region between helix A and B.

Interestingly, solvent accessible surface area values (ASA; see Materials and Methods section for details) calculated for the crystallographic structures of Cre and Tre (*i.e.* PDB ID 1Q3U and 5U91, respectively), which do not contain the Nt tail, show that in the crystal structure of Tre ASA values for residues forming the hydrophobic V-shaped conserved region between helix A and B (*i.e.* 27, 30, 31, 42, 45, 46, 49 and 63) are much higher than for Cre, indicating that this hydrophobic region is much more exposed in Tre than in Cre. However, for the conformations of Tre including the Nt tail obtained from MD, those ASA values drop to numbers equivalent to the ones obtained for the crystal structure of Cre, which does not include the Nt tail (Table S2). These results indicate that the packing of the Nt in Tre reduces the ASA to similar values obtained for Cre without the Nt tail, suggesting that evolution may have found a way to stabilize the evolved protein Tre by reducing its hydrophobic solvent accessibility with the help of the structural organization of its Nt tail.

These findings further support the hypothesis in which the evolutionary changes needed to obtain new DNA target specificity, which may generate certain instability, might trade off during the evolutive process [52], [53], [54] with stabilizing mutations introduced at the Nt tail. As mentioned above, evolutionary simulations investigating the consequences of marginal thermostability in proteins have indicated that the natural tendency of proteins toward marginal stability, and the range of stabilities occurring during evolution, may have a significant effect on the evolutionary process [9], [58]

## 3 Conclusions

In this work, we investigate how evolution and conformational dynamics promote disordered regions from being function-dispensable to become essential for the enzymatic activity of evolved SSRs. Our studies comparing wild-type Cre and the evolved Tre recombinase rationalize evolutive data by revealing a dynamic ensemble of conformational states of their N-terminal tails that may determine diverse functional consequences. We propose new plausible molecular mechanistic insights into disorder-function relationships explaining the loss of function in Tre upon the deletion of its Nt tail. We predict that, opposite to Cre in which the Nt tail appears unstructured and is irrelevant for its function, the structural organization and packing of the N-terminal tail of Tre translates into a gain of stability, which we hypothesize might be the reason for its essential role in the evolved recombinase. Our work suggests the SSR Cre/loxP and related evolved systems as a new attractive exemplary model to investigate functional molecular mechanisms of IDRs. Furthermore, our findings highlight the potential of N-terminal tails to be exploited for regulation of the activity of Cre-like SSRs and evidence an important add-on for protein engineering since they offer a new tool to the bioengineering repertoire for the future design of SSRs not explored until now and of relevance for their use in biotechnology and genomic medicine.

## 4 Materials and Methods

### 4.1 Recombination assays in *E. coli.*

For expression in *E. coli*, Cre and Cre-ΔNt_12_ were cloned into the pEVOloxP vector, while Tre and Tre-ΔNt_12_ were cloned into pEVOloxLTR [25] utilizing the unique BsrGI and XbaI (NEB, Ipswich, MA, USA) restriction sites. Brec1 and Brec1-ΔNt_12_ were cloned into pEVOloxBTR [26]. For the Nt deletion of 12 amino acids (ΔNt_12_), Cre, Tre, and Brec1 recombinases were PCR-amplified using respective primers and Phusion High Fidelity DNA Polymerase (NEB, Ipswich, MA, USA). Expression of the recombinases from the pBAD promoter was induced with L-(+)-arabinose (Sigma-Aldrich Chemie GmbH). Single colonies of XL1-blue *E.coli* (recA1 endA1 gyrA96 thi-1 hsdR17 supE44 relA1 lac [F ´ proABlacIqZΔM15 Tn10 (Tetr)]; Agilent, Santa Clara, CA, USA) containing pEVO plasmids harboring the respective recombinases were cultured overnight in 5 ml Luria broth (LB) medium with 30 μg/ml chloramphenicol and 0, 10 or 100 μg/ml L-(+)-arabinose at 37 °C and 200 rpm. Subsequently, plasmid extraction (Qiagen, Hilden, Germany), digestion and gel electrophoresis were performed to detect recombination as previously described [64]. To quantify recombinase activities, band intensities were determined with GelAnalyzer 19.1 (GelAnalyzer 19.1 (www.gelanalyzer.com)) using the ladder bands as a point of reference. Recombination rates were calculated as a percentage of the sum of all the bands in that lane.

### 4.2 Sequence- and AI-based conformational predictions.

Blast (blastp) [39] was used to look for sequence homology between the first 20 residues of Cre and Tre and proteins of known structure in the PDB (https://www.rcsb.org/). The hits with highest sequence homology and no gaps in this region were selected for structural analysis. Secondary structure predictions were carried out with the webservers CFSSP [40] and PSIPRED [41]. AlphaFold [42], [43] predictions were carried out with the first 35 residues of Cre and Tre, which include the Nt tail and helix A. Sequence data from residues 1 to 35 in FASTA format was used as input. Five models were obtained for each sequence as default option. The AlphaFold (v2.0) source was cloned (*date 20*–*10-2021*) and deployed following all instructions in the *readme* file. Genetic sequence and structural databases were obtained in full and stored locally using NVMe SSD drives (Crucial CT2000P2SSD8) for faster genetic search performance. Docker (version 20.10.7) was used to build the application container. Python wrappers “*hhblits.py*” and “*jackhmmer.py*” were modified and set to use all available CPUs. The preset used was *casp14*, which includes all genetic databases and 8 ensembles. The folding experiments were deployed on a SuperMicro server equipped with 192 Gb of RAM, 72 Intel Xeon Gold cores (3.1Ghz) and 10 × RTX 2080 Ti GPU cards. At the time of running, the server was using Ubuntu 20.04.3, Kernel 5.4.0–89, Nvidia driver 470.74 and CUDA 11.4.

### 4.3 Molecular modeling of Nt_20__._

The crystallographic structures of the Cre and Tre recombinases in complex with their respective DNA target site (loxP and loxLTR, respectively) used for our studies were extracted from the PDB (PDB ID 1Q3U for Cre/loxP [21] and PDB ID 5U91 for Tre/loxLTR [19]). The MOE software [65] was used to model the Nt_20_ in each monomer of Cre and Tre in extended conformation.

### 4.4 ST MD simulations.

Each of the recombinase/DNA crystallographic structures containing their corresponding modeled Nt_20_ in extended conformation were placed in a dodecahedron box sufficiently large to contain the recombinase/DNA complex surrounded by solvent. Parameters for the protein and DNA were assigned using the ff03ws [66] and parmbsc1 [67] force fields, respectively. Each protein/DNA complex was solvated with TIP4P/2005 water molecules [68], and Na+/Cl- ions were added to the system in order to maintain charge neutrality. All simulations were conducted under periodic boundary conditions using the GROMACS software suite (version 2018.3) [44], [45]. Long-range electrostatics were handled using the particle mesh Ewald (PME) method [69] with a cut-off of 1.2 nm. LINCS [70] was used to constraint the bonds involving hydrogens. The pressure of the system was set to 1 bar using the Parrinello-Rahman barostat (τ = 5 ps) [71]. The temperature was maintained at 300 K using the velocity rescaling method (τ = 2 ps) [72]. For each recombinase/DNA system, an initial steepest descent minimization of the solvent was performed with restraints on the solute followed by a short equilibration for 50 ps with the NPT ensemble at 300 K. A series of minimizations were then performed with and then without restraints on the solute for 2 ns, followed by an equilibration step of 50 ns in an NPT ensemble. A simulated tempering (ST) MD approach [46] was used to ensure enhanced sampling of the disordered Nt region. In these simulations, for each recombinase/DNA complex, residues 1 to 20 of each protein monomer were kept unrestrained, and the rest of the protein residues (*i.e.* 21–341) and the DNA were restrained with a harmonic potential. The temperature of the system was changed periodically from 300 to 450 K. The Metropolis algorithm [73] was used to control temperature variations. To begin the ST MD simulation, the weights were initially set according to the Park and Pande procedure [74] and systematically updated according to the Wang-Landau adaptive weighting scheme [75]. The production run was carried out for 1.3 μs for each system.

*Structural analysis.*

The structural analysis of the MD trajectories was carried out using the VMD software [47]. MD-based secondary structure analysis was carried out with DSSP [48], [50].

*Clustering analyses and selection of representative configurations.*

The complete ST MD trajectories obtained for the Cre/loxP and Tre/loxLTR systems were used as input. The configurations for the clustering analysis were taken from the ensemble at 300 K of the unrestrained Nt_20_ for each of the four protein monomers. These configurations were then grouped in sub-ensembles and clustered using the GROMOS clustering method [45], [76]. The cutoff was selected based on RMSD criteria. The topmost populated Nt_20_ configuration was selected as representative for each of the four monomers of Cre and Tre.

### 4.5 Modelling of protein-DNA complexes for classical MD simulations.

The MOE software [65] was used to generate the full-recombinase structure (*i.e.* residues 1–341). The Nt_20_ representative conformations obtained from the ST MD trajectories were linked to residue 21 of the respective protein monomers of the crystal structure of each recombinase/DNA system based on structural overlapping. The resulting full complexes Cre_1-341_/loxP and Tre_1-341_/loxLTR were refined by classical MD simulations.

### 4.6 Classical MD simulations.

The Cre_1-341_/loxP and Tre_1-341_/loxLTR complexes were refined by classical MD using the same setup as for the ST MD simulations. The temperature was maintained at 300 K using the stochastic velocity rescaling method [72]. The pressure was set to 1 bar using the Parrinello-Rahman barostat [71]. A series of minimizations were then conducted using the steepest descent method restraining the solute (protein and DNA) first, and then simulating without restrains for 2 ns. The system was further subjected to equilibration for 50 ns in an NPT ensemble at 300 K. The production run was carried out at a constant temperature of 300 K for 500 ns on both systems. Positional restraints were applied on the Cα atoms of the interacting pair Arg32-Glu69 present at the monomers interface (*i.e.* residue 32 of Tre_A_ forming a salt bridge with residue 69 of Tre_D_ (Fig. S5)). Without these Cα-Cα restraints, during the simulations the protein monomers tend to move apart from each other resulting in a loosened destabilized intermonomer interface.

*Clustering analysis of the classical MD simulations.*

For each recombinase system, a cumulative trajectory from the classical MD simulations was generated by extracting the NTD of the four individual MD trajectories for each of the respective protein monomers, which were then concatenated to perform the clustering analysis. GROMOS clustering method was used [76]. The cutoff was selected based on RMSD criteria.

### 4.7 *In silico* evolution calculations.

Monomers A to D were considered separately and taken from the representative Tre structure obtained from our ST-MD simulations. PyMOL version 2.4 (Schrödinger LLC; https://pymol.org) was used for visualization and to prepare the structure of the 4 individual protein monomers. Positions 7, 9, 10, 16, and 30 of each protein monomer were mutated to every amino acid of the 20 canonical, yielding every possible sequence permutation (*i.e.* 20^5^ = 3.2 million sequences). The newly created mutant sequences were stored in FASTA format, and their respective 3D structure was modeled with Modeller version 9.24 [77] using each individual monomer of Tre as template. This resulted in 12.8 million decoy structures (*i. e.* 3.2 million sequences × 4 monomers).

The Rosetta Relax application [59] from the Rosetta software suite (version 3.3, www.rosettacommons.org) [60] was used to score each decoy using the ref2015 scoring weights, default parameters and fixed backbone settings. Rosetta 3 relax binaries and shared libraries were compiled from source using the GNU gcc compiler and used to score structures. The Rosetta score is a combination of physics-based and statistics-based potentials. Rosetta uses two energy functions that fluctuate upon how detailed the sampling is being done. A coarse-grained representation model is used to speed up early stage sampling, while a more expensive all-atom model is used for middle and final stage samplings [78]. The functions that calculate coarse-grained sampling are fundamentally different from those used in full atom sampling. Difficulties arise when reconciling both functions, therefore, a composite value is used to represent them simultaneously. This value is called “Rosetta Energy Units” (REU).

Per-residue energies were calculated for positions 1 to 33. The rest of the protein (positions 34 to 341) were excluded from the scoring. Per-residue energies of each decoy were scavenged and stored in a MySQL database for later processing. The histogram built for each monomer to inspect the overall energy distribution and the frequency of favorable mutations used an energy-bucket size of 10 ΔG REU, which was chosen based on the following criteria: *i)* the average margin of error of the Rosetta score, *ii)* the numerical distance between the best and worse decoys, *iii)* the score variance (across the entire population of decoys), *iv)* the total amount of decoys per monomer.

For this high throughput *in-silico* evolution framework, and in order to automate calculations and assemble data, we established an in-house computational pipeline (to be published elsewhere). Python3, Shellscript and SQL were used as programming languages. The Romeo partition of the Taurus HPC [79] was used to deploy parallel Rosetta scoring jobs. A total of 4 million CPU hours were invested in the scoring of decoys.

### 4.8 Thermodynamic stability calculations.

Thermodynamic stability calculations were performed using the Rosetta software suite (version 3.3, www.rosettacommons.org) [60] and the NTD structures of Cre and Tre. The crystal structures of Cre and Tre (PDB ID 1Q3U [21] and 5U91 [19], respectively), which lack the Nt tail, were used for the calculations excluding the Nt_20_ region (Cre-ΔNt_20_, Tre-ΔNt_20_; residues 21 to 129 of NTD). Calculations were performed for each monomer of the crystal structure. The representative structures of the top 10 most populated clusters obtained from the clustering analysis of the cumulative trajectories of the MD simulations were used for the calculations including the Nt_20_ (Cre+Nt_20_, Tre+Nt_20_; residues 1 to 129). These calculations were also carried out for each of the monomers and resulted in a total of 168 structures. Each structure was minimized using the Rosetta *score12_cst* function and processed with the Rosetta *ddg_monomer* protocol to compute stability energies (*wildtype_dg*, ΔG). The provided Rosetta online documentation with the necessary steps (https://www.rosettacommons.org/docs/latest/application_documentation/analysis/ddg-monomer) was used for additional preparation of input structures. The protocol parameters were based on the work of Nisthal *et al.* [63]. We followed the option referred to as the “Somemin parameter set“, which has shown the most accurate stability predictions in benchmarking of protein stability-prediction algorithms, as reported by Kellogg *et al*. [61]. Python scripting was used to iteratively scavenge the resulting values of *wildtype_dG* across several hundreds of output files. All protocols were executed in a sysGen SuperMicro A+ server equipped with 256 Gb of RAM, 128 AMD EPYC cores (2,0 Ghz) and 2 TB of SDD.

### 4.9 Solvent-accessible surface area (ASA) calculations.

ASA values were calculated using Discovery Studio 2021 v21.1.0.20298 (BIOVIA, Dassault Systems, San Diego, 2021) with default settings, 240 grid points per atom and a probe radius of 1.40 Å.

## CRediT authorship contribution statement

**Carla Guillén-Pingarrón:** Conceptualization, Methodology, Software, Data curation, Formal analysis, Validation, Investigation, Visualization, Writing – original draft, Writing – review & editing. **Pedro M. Guillem-Gloria:** Conceptualization, Methodology, Software, Data curation, Formal analysis, Validation, Investigation, Visualization, Writing – original draft, Writing – review & editing. **Anjali Soni:** Conceptualization, Methodology, Formal analysis, Investigation, Visualization, Writing – original draft. **Gloria Ruiz-Gómez:** Conceptualization, Methodology, Supervision, Writing – review & editing. **Martina Augsburg:** Methodology, Investigation, Visualization. **Frank Buchholz:** Conceptualization, Visualization, Supervision, Project administration, Funding acquisition, Writing – original draft, Writing – review & editing. **Massimiliano Anselmi:** Conceptualization, Methodology, Supervision, Writing – review & editing. **M. Teresa Pisabarro:** Conceptualization, Visualization, Supervision, Project administration, Funding acquisition, Writing – original draft, Writing – review & editing.

## Declaration of Competing Interest

The authors declare that they have no known competing financial interests or personal relationships that could have appeared to influence the work reported in this paper.
