# Supplementary material for: Conformational dynamics promotes disordered regions from function-dispensable to essential in evolved site-specific DNA recombinases
Source: Comput Struct Biotechnol J. 2022 Jan 22;20:989–1001. doi: 10.1016/j.csbj.2022.01.010 (PMC8860914; doi:10.1016/j.csbj.2022.01.010)
Supplement: Supplementary data 1 [file mmc1.docx]

**SUPPLEMENTARY INFORMATION**

**Conformational dynamics promotes disordered regions from function-dispensable to essential in evolved site-specific DNA recombinases**

**Carla Guillén-Pingarrón^a,‡^, Pedro M. Guillem-Gloria^a,‡^, Anjali Soni^a,‡^, Gloria Ruiz-Gómez^a^, Martina Augsburg^b^, Frank Buchholz^b^, Massimiliano Anselmi^c,*^, M. Teresa Pisabarro^a,*^**

^a^Structural Bioinformatics, BIOTEC, TU Dresden, Tatzberg 47-51, 01307 Dresden, Germany. ^b^University Carl Gustav Carus and Medical Faculty, UCC, Medical Systems Biology, TU Dresden, Fetscherstrasse 74, Dresden, Germany.

^c^Theoretical Physics and Center for Biophysics, Saarland University, 66123 Saarbrücken, Germany.

**^‡^** These authors contributed equally

***** Corresponding authors:

MA: manselm@gwdg.de; Phone: +49 681 3023959

MTP: [maria_teresa.pisabarro@tu-dresden.de](mailto:maria_teresa.pisabarro@tu-dresden.de); Phone: +49 351 46340071

1...| ....|....|....|....|....|....|....|....|....|....|...

Cre MSNLL--T**V**H**QN**LPALP**V**DATSDEVRKNLMD**M**FRDR**Q**AFSEHTW**K**MLLSVCRSWAAWCKL 58

Tre MSNLL--T**L**H**HS**LPALP**A**DATSDEVRKNLMD**V**FRDR**P**AFSEHTW**E**MLLSVCRSWAAWCKL 58

Brec1 MSILL--T**L**H**QS**LSALL**V**DATSDEARKNLMD**V**LRDRQAFSERTWKVLLSVCRTWAAWCKL 58

Dre MSELIISGSSGGFLRNI-GKEYQEAAENFMRFMNDQGAYAPNTLRDLRLVFHSWARWCHA 59

Panto MKHLA-QIKTGALPAELTQLTPEDIANNLRRFIADKAAYSENTFRDLLSVIRRWAFWCNE 59

*. * : :: :*: .: *: *:: .* . * * : ** **:

Cre NNRKWFPAEPEDVRDYLL**Y**LQARGLAVKTIQQHL**GQ**LNMLHRRSGLPRPSDSNAVSLVMR 118

Tre NNRKWFPAEPEDVRDYLL**H**LQARGLAVKTIQQHL**CR**LNMLHRRSGLPRPSDSNAVSLVMR 118

Brec1 NNRKWFPAEPEDVRDYLLHLQARGLAVNTILQHLAQLNMLHRRFGLPRPGDSDAVSLVMR 118

Dre RQLAWFPISPEMAREYFLQLHDADLASTTIDKHYAMLNMLLSHCGLPPLSDDKSVSLAMR 119

Panto RDVGYLPIDPELAREYFLQMAESGLASSTIDKHYAMMNMLCRESGLPDLRGSVDLKRSMK 119

.: ::* .** .*:*:* : .** .** :* :*** . *** .. :. *:

Cre RIRKENV-DAGER**A**KQALAFERTDFDQVRSLMENSDRCQDIRNLAFLG**I**AYNTLLRIAEI 177

Tre RIRKENV-DAGER**T**KQALAFERTDFDQVRSLMENSDRCQDIRNLAFLG**V**AYNTLLRIAEI 177

Brec1 RIRRENV-DAGERTKQALAFERTDFDQVRALMENSERGQDIRTLALLGVAYNTLLRVSEI 177

Dre RIRREAATEKGERTGQAIPLRWDDLKLLDVLLSRSERLVDLRNRAFLFVAYNTLMRMSEI 179

Panto RIRREAV-LQGERTGQAVPFRLPDLQLLSHLMGRSDRLTDQRNLAFLFVAYNTLCRMSEL 178

***:* . ***: **: :. *:. : *: .*:* * *. *:* :***** *::*:

Cre ARIRVKDISRTDGGRMLIHIGRTKTLVSTAGVEKALSLGVTKLVERWISVSGVADDPNNY 237

Tre ARIRVKDISRTDGGRMLIHIGRTKTLVSTAGVEKALSLGVTKLVERWISVSGVADDPNNY 237

Brec1 ARIRIKDISRTDGGRMLIHISRTKTLVSTAGVEKALSLGVTKLVERWISVSGVASDPNNY 237

Dre SRIRVGDLDQTG-DTVTLHISHTKTITTAAGLDKVLSRRTTAVLNDWLDVSGLREHPDAV 238

Panto SRIRVRDLDISDSGHVVINLSHTKTMVTAAGVIKHLSRAAAGHLMHWLELSGLIHHPDAM 238

:***: *:. :. . : :::.:***:.::**: * ** .: : *:.:**: .*:

Cre LFCRVR**KN**GVAAPSATSQLST**R**AL**EG**IFEATHRLIYG-AKDDSGQRYLAWSGHSARVGAA 296

Tre LFCRVR**RY**GVAAPSATSQLST**Y**AL**QR**IFEATHRLIYG-AKDDSGQRYLAWSGHSARVGAA 296

Brec1 LFCQVRINGVAVPSATSRLSTDVLRKIFEAAHRLIYG-AKDGSGQRYLAWSGHSARVGAA 296

Dre LFPPIHRSNKARIT-TTPLTAPAMEKIFSDAWVLLNKRDATPNKGRYRTWTGHSARVGAA 297

Panto VFGPVRHNNTAGVS-EKPMSAPATEKIFKDAWDLLGKEPVQDNKGRYAKWSGHSARVGAA 297

:* :: . * : . ::: . . **. : *: . ** *:*********

Cre RDMARAGVSIPEIMQAGGWT**N**VN**I**VMNYIRNLDSETGAMVRLLEDGD 343

Tre RDMARAGVSIPEIMQAGGWT**T**VN**S**VMNYIRNLDSETGAMVRLLEDGD 343

Brec1 RDMARAGVSIAEIMQAGGWTTVESVMNYIRNLDSETGAMVRLLEDGD 343

Dre IDMAEKQVSMVEIMQEGTWKKPETLMRYLRRGGVSVGANSRLMDS-- 342

Panto MDMAERDATITQIMQEGTWQDPKTVMRYLRRSESQKGKMSGILDGE- 343

***. .:: :*** * * : :*.*:*. . * :::.

Figure S1. Multiple sequence alignment of Cre, the evolved recombinases Tre and Brec1, and other Cre-like SSRs. The 19 mutations introduced by the evolution of Tre from Cre are highlighted in bold, and those at the Nt tail are shown following the color code used in the molecular models showed in the figures in the main text. The residues forming the “V-shaped” hydrophobic surface between helices A and B are highlighted with a gray box. The alignment was obtained with Clustal O (v 1.2.4).

**
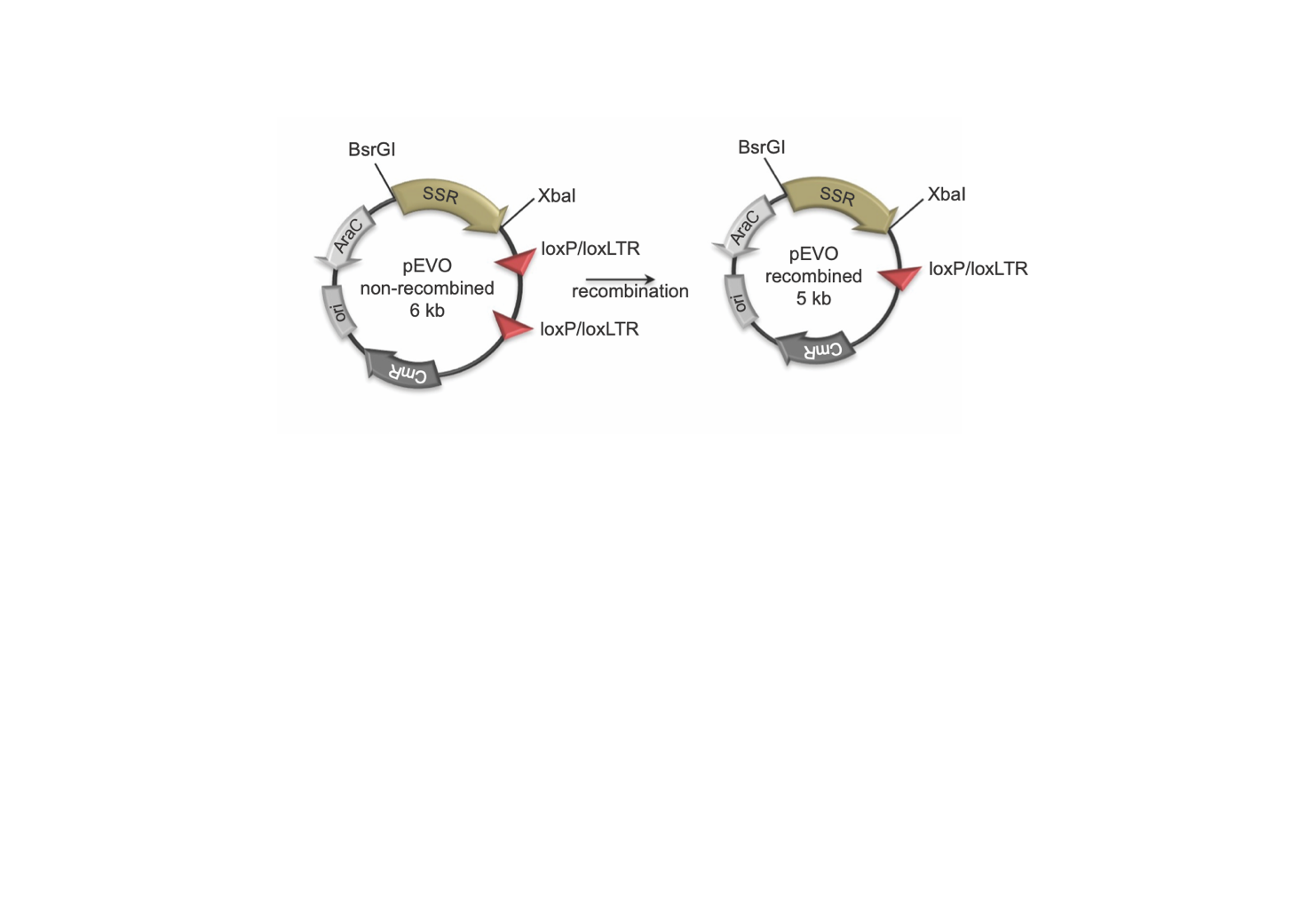
**

Figure S2. Schematic drawing of the plasmid assay. Important regions in the plasmids are indicated. Note the reduced size of the plasmid after recombination. The restrictions sites (BsrGI and XbaI) used for cloning the recombinases (SSR) are depicted. The *loxP/loxLTR* target sites are shown as red triangles. CmR, chloramphenicol resistance gene; ori, origin of replication; AraC, arabinose operon regulatory gene.


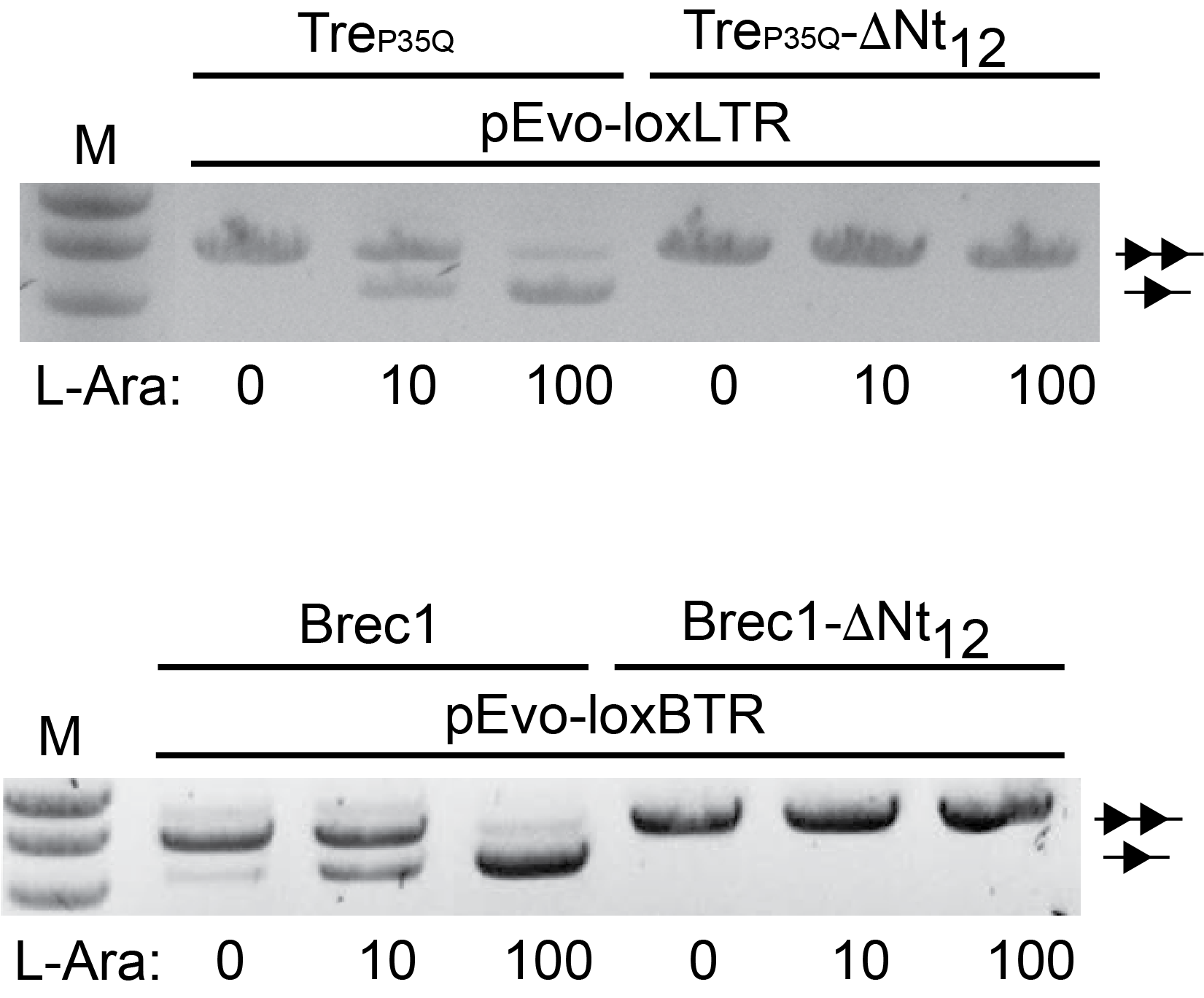


|  | **Rec [%]** | | | |
| --- | --- | --- | --- | --- |
| **L-Ara** | **Tre_P35Q_** | **Tre_P35Q_-ΔNt_12_** | **Brec1** | **Brec1-ΔNt_12_** |
| 0 | 0 | 0 | 10 | 0 |
| 10 | 35 | 0 | 33 | 0 |
| 100 | 89 | 0 | 92 | 0 |

Figure S3. Recombination efficiency of Tre_P35Q_/loxLTR and Brec1/loxLTR recombinase systems and their respective 12 residue N-terminal deletion mutants (indicated by ΔNt_12_). Employed L-arabinose levels (in mg/ml) to induce recombinase expression are displayed below the gel. The line with two triangles and one triangle depict the band sizes for non-recombined and recombined plasmids, respectively. M = marker (1 kb ladder). BOTTOM: Quantification of recombination efficiencies in *E.coli*. Recombination (in %) was calculated from measuring gel band intensities for indicated recombinases.


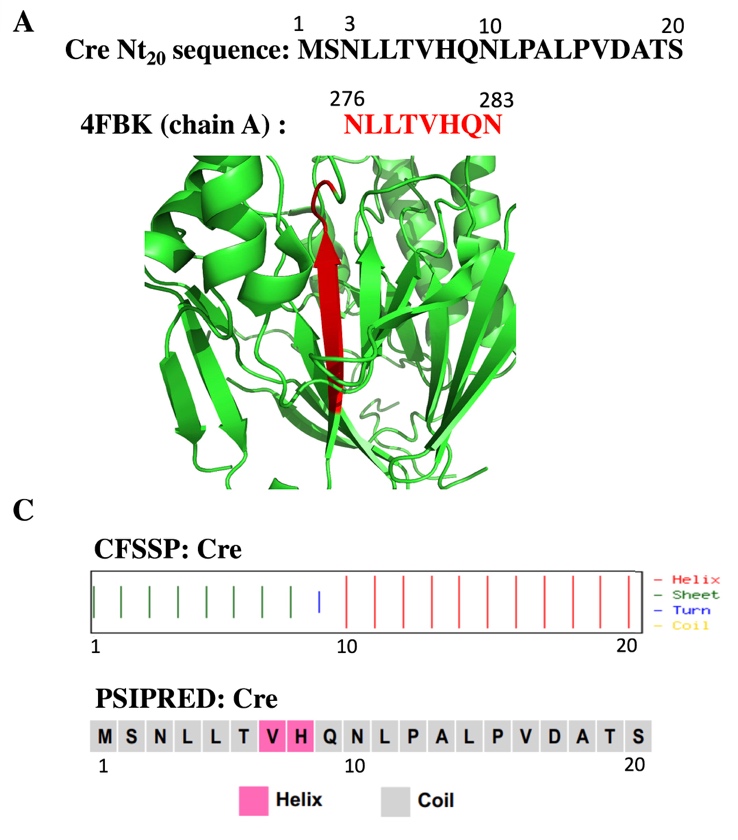

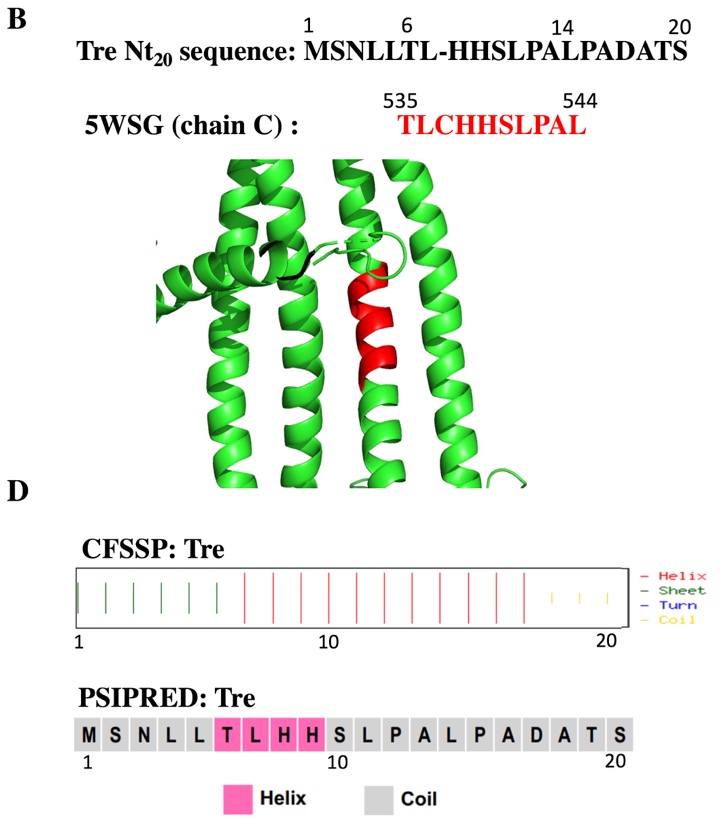


**
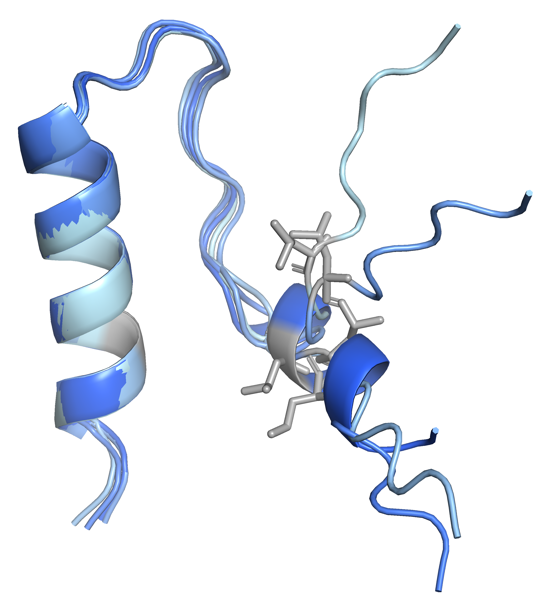

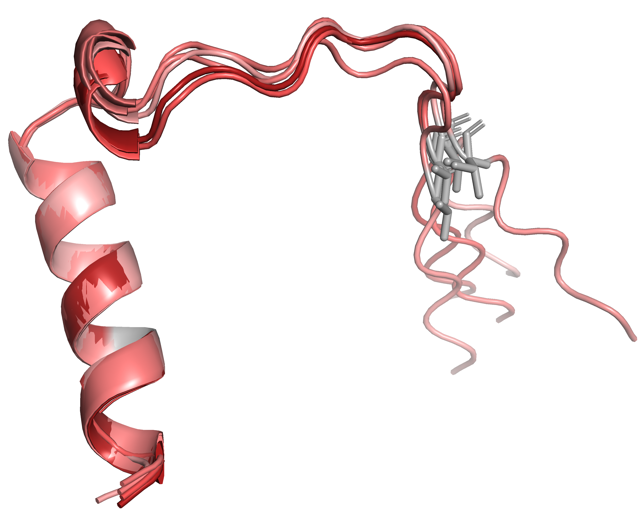
E** **F**


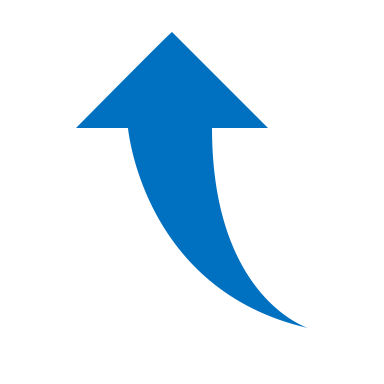

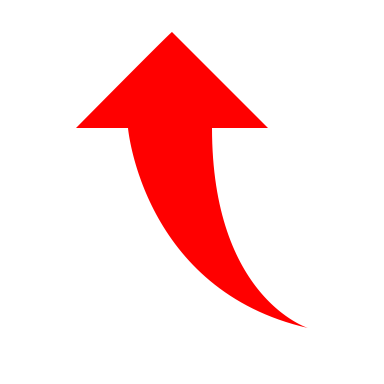


**Helix A**

**Helix A**

Figure S4. Sequence-based analysis of the intrinsic conformational propensities of the N-terminal tail of Cre and Tre. A-B) Top sequence similarities found in the Protein Data Bank (PDB) for Cre-Nt_20_ and Tre-Nt_20_. A) Sequence alignment showing the best Blastp results obtained for Cre-Nt_20_ and PDB ID 4FBK (DNA repair and telomere mainteinance protein Nbs1) shown at the top. The structure of PDB ID 4FBK is displayed in a green cartoon with the sequence-homologous region in β-strand conformation highlighted in red. B) Sequence alignment showing the Blastp results obtained for Tre-Nt_20_ and PDB ID 5WSG (Pre-mRNA-splicing factor 8) shown at the top. The structure of PDB ID 5WSG is displayed in a green cartoon with the sequence-homologous region in helical conformation highlighted in red. C-D) Secondary structure prediction for Cre-Nt_20_ and Tre-Nt_20_ using the CFSSP (top) and the PSIPRED (bottom) web servers. E-F) AlphaFold folding prediction results obtained for the first 35 residues (*i.e.* helix A and N-terminal tail) of Cre- (red) and Tre (blue). Top-five ensembles are shown in a gradient color indicating the darker the better. The N-terminal is indicated by an arrow. Side chains of residues in position 7 are shown in gray sticks.


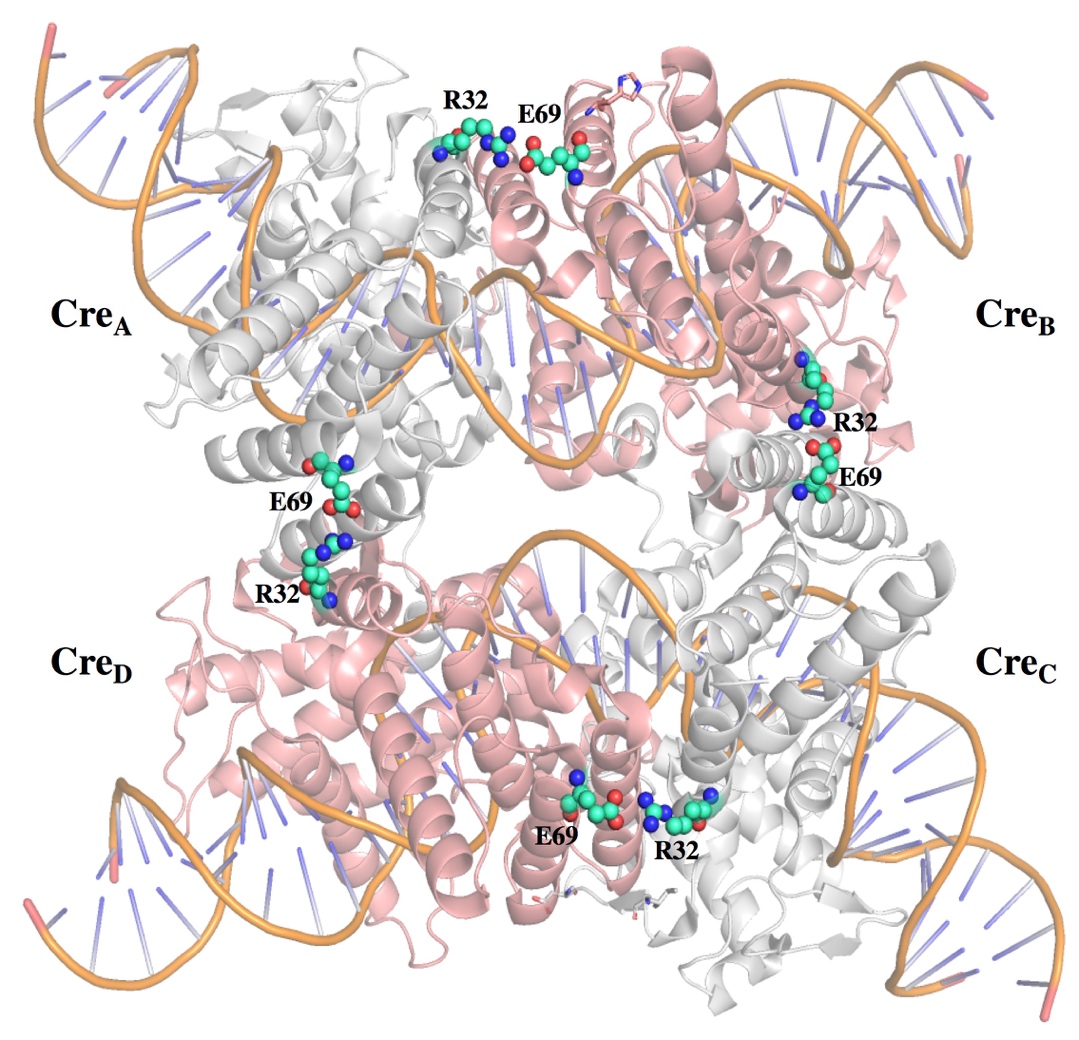


Figure S5. Structure of the Cre/loxP complex (PDB ID 1Q3U) showing the spatial location of residues Arg32 and Glu69 (in atom-colored CPK spheres) in each monomer-monomer interface. The protein monomers in active conformation are shown in a pink ribbon and the ones in inactive conformation in white. The DNA backbone is shown as a yellow ribbon with the bases in ladder representation.


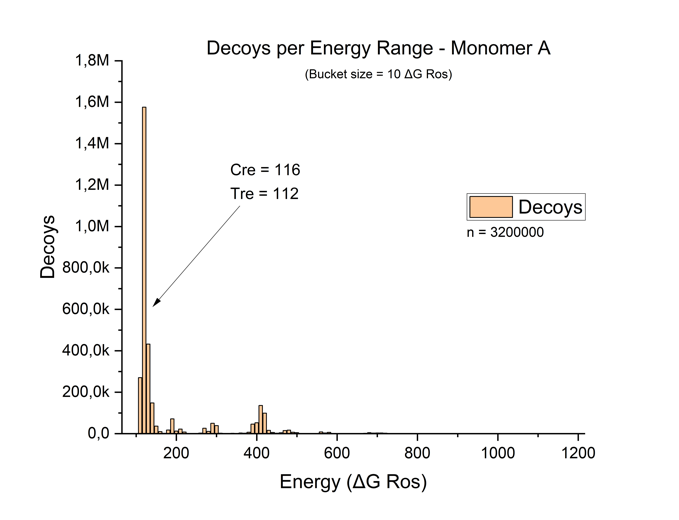

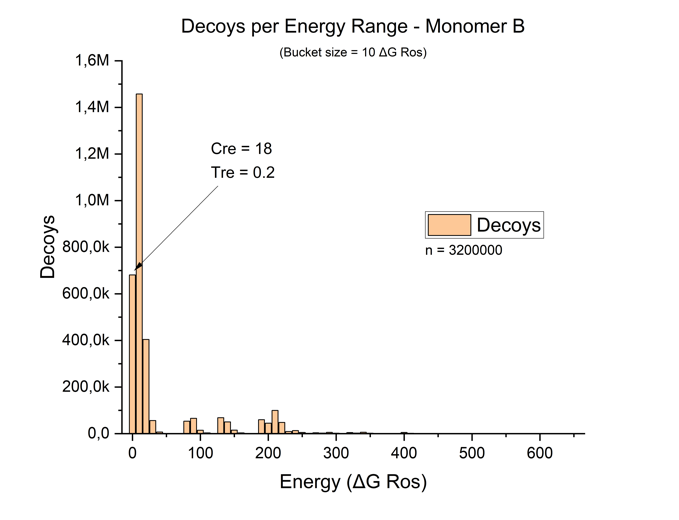


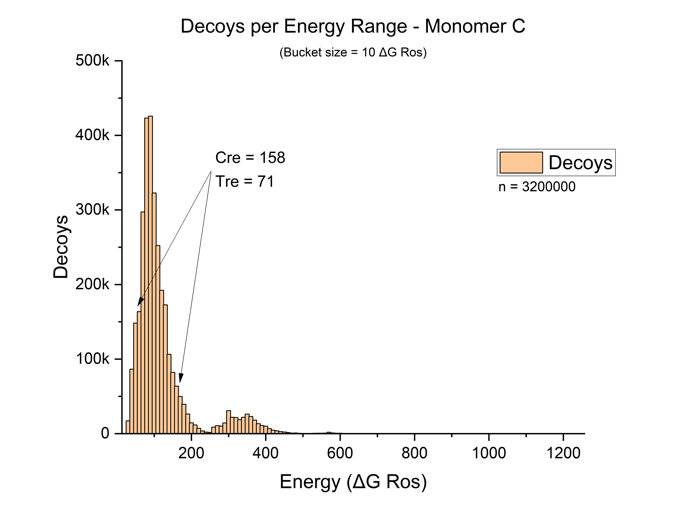

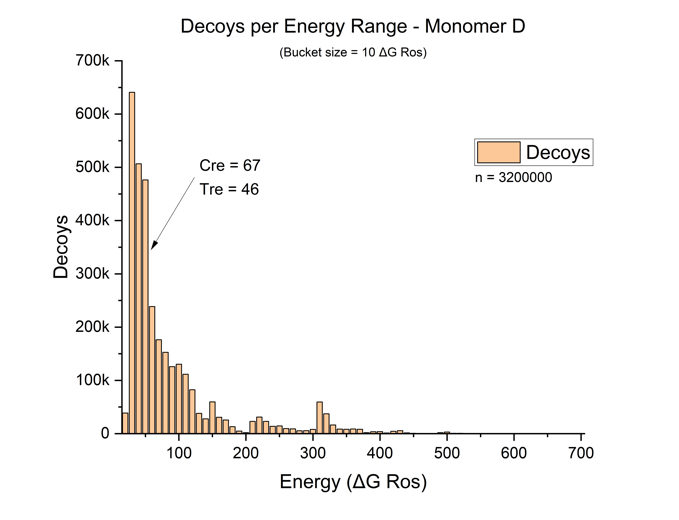


Figure S6. Decoy energy distribution in monomers Tre_A_, Tre_B_, Tre_C_ and Tre_D_ (n = 3.2 million decoys per monomer). Each bin (orange boxes) represents total decoys grouped in buckets of 10 ΔG Ros (lower energy denotes better scores). Energy scores obtained for Cre and Tre sequences are shown as reference, and an arrow is used to point to the bin in which they appear.

Table S1. Energy reference values per monomer for Cre and Tre sequences and size of “best decoys” subset.

|  | | **Rosetta Energy (ΔG Ros)** | | | | | | **Size of  “best decoys” subset** |
| --- | --- | --- | --- | --- | --- | --- | --- | --- |
| **Protein Monomer** | **Cre** | **Tre** | **AVG^a^** | **STD^b^** | **MIN^c^** | **MAX^d^** |  |  |
| **A** | 116 | 112 | 178 | 117 | 71 | 1212 | 393.525 |  |
| **B** | 18 | 0,2 | 40 | 70 | -7 | 658 | 2.335.456 |  |
| **C** | 158 | 71 | 121 | 80 | 21 | 1248 | 2.710.979 |  |
| **D** | 67 | 46 | 86 | 81 | 17 | 699 | 1.954.801 |  |

^a^Average energy value. ^b^Standard deviation value. ^c^Minimun energy value. ^d^Maximun energy value.

Table S2. Sidechain Solvent Accessibility (ASA) calculated for the crystallographic structures of Cre (PDB ID 1Q3U) and Tre (PDB ID 5U91), which do not include the Nt tail, and for the predicted Tre_A_ and Tre_D_ conformations without and including the Nt_12_ tail. Values are shown in percentage. Calculations were made with Discovery Studio 2021 v21.1.0.20298 (BIOVIA, Dassault Systemes, San Diego, 2021) with default settings, 240 grid points per atom and a probe radius of 1.40Å.

| **Residue** | **Cre**  **X-ray** | **Residue** | **Tre**  **X-ray** | **Tre_A_**  **MD**  **(-Nt_12_)** | **Tre_D_**  **MD**  **(-Nt_12_)** | **Tre_A_**  **MD**  **(+Nt_12_)** | **Tre_D_**  **MD**  **(+Nt_12_)** |
| --- | --- | --- | --- | --- | --- | --- | --- |
| LEU27 | 11,65 | LEU27 | 46,58 | 49,62 | 38,48 | 14,177 | 16,71 |
| MET30 | 0 | VAL30 | 9,16 | 13,162 | 13,16 | 9,728 | 13,16 |
| PHE31 | 32,43 | PHE31 | 72,63 | 89,189 | 61,82 | 20,608 | 11,49 |
| TRP42 | 14,46 | TRP42 | 17,88 | 13,149 | 10,51 | 1,315 | 6,57 |
| LEU45 | 1,01 | LEU45 | 14,18 | 14,177 | 8,10 | 4,051 | 5,06 |
| LEU46 | 32,91 | LEU46 | 63,80 | 96,709 | 67,34 | 10,633 | 13,16 |
| CYS49 | 0 | CYS49 | 20,45 | 20,671 | 8,84 | 1,3 | 0,74 |
| TRP63 | 13,88 | TRP63 | 24,87 | 60,015 | 19,30 | 25,733 | 17,99 |

Table S3. Frequencies and normalized values obtained for residue-pair combinations in positions 7 and 30 for each Tre monomer. The highest value for each monomer is highlighted in bold. For comparison, the L7-V30 interacting pair of Tre and the V7-M30 interaction pair of Cre are highlighted in blue and red, respectively.

| **Monomer A** | | | |  | **Monomer B** | | | |
| --- | --- | --- | --- | --- | --- | --- | --- | --- |
| **Pos7** | **Pos30** | **Frequency** | **Normalized** |  | **Pos7** | **Pos30** | **Frequency** | **Normalized** |
| A | A | 6806 | 0,993 |  | A | A | 7021 | 0,993 |
| A | I | 6730 | 0,982 |  | A | I | 5522 | 0,781 |
| A | L | 6833 | 0,997 |  | A | L | 5707 | 0,807 |
| A | M | 6816 | 0,995 |  | A | M | 6879 | 0,973 |
| A | F | 5588 | 0,816 |  | A | F | 7042 | 0,996 |
| A | W | 5198 | 0,759 |  | A | W | 6962 | 0,985 |
| A | V | 6842 | 0,999 |  | **A** | **V** | **7071** | **1,000** |
| I | A | 6793 | 0,991 |  | I | A | 6568 | 0,929 |
| I | I | 6741 | 0,984 |  | I | I | 3185 | 0,450 |
| I | L | 6818 | 0,995 |  | I | L | 3357 | 0,475 |
| I | M | 6798 | 0,992 |  | I | M | 6249 | 0,884 |
| I | F | 5143 | 0,751 |  | I | F | 6616 | 0,936 |
| I | W | 5105 | 0,745 |  | I | W | 6433 | 0,910 |
| I | V | 6828 | 0,996 |  | I | V | 6661 | 0,942 |
| L | A | 6830 | 0,997 |  | L | A | 6877 | 0,973 |
| L | I | 6836 | 0,998 |  | L | I | 4595 | 0,650 |
| L | L | 6843 | 0,999 |  | L | L | 4851 | 0,686 |
| L | M | 6821 | 0,995 |  | L | M | 6642 | 0,939 |
| L | F | 6270 | 0,915 |  | L | F | 6898 | 0,976 |
| L | W | 5989 | 0,874 |  | L | W | 6803 | 0,962 |
| L | V | 6832 | 0,997 |  | L | V | 6933 | 0,980 |
| M | A | 6813 | 0,994 |  | M | A | 6706 | 0,948 |
| M | I | 6831 | 0,997 |  | M | I | 3769 | 0,533 |
| **M** | **L** | **6852** | **1,000** |  | M | L | 3996 | 0,565 |
| M | M | 6828 | 0,996 |  | M | M | 6408 | 0,906 |
| M | F | 6424 | 0,938 |  | M | F | 6747 | 0,954 |
| M | W | 5107 | 0,745 |  | M | W | 6631 | 0,938 |
| M | V | 6842 | 0,999 |  | M | V | 6799 | 0,962 |
| F | A | 1970 | 0,288 |  | F | A | 6373 | 0,901 |
| F | I | 3416 | 0,499 |  | F | I | 2275 | 0,322 |
| F | L | 4422 | 0,645 |  | F | L | 2461 | 0,348 |
| F | M | 4981 | 0,727 |  | F | M | 5931 | 0,839 |
| F | F | 850 | 0,124 |  | F | F | 6408 | 0,906 |
| F | W | 976 | 0,142 |  | F | W | 6217 | 0,879 |
| F | V | 4491 | 0,655 |  | F | V | 6478 | 0,916 |
| W | A | 6543 | 0,955 |  | W | A | 6305 | 0,892 |
| W | I | 5918 | 0,864 |  | W | I | 2190 | 0,310 |
| W | L | 6751 | 0,985 |  | W | L | 2057 | 0,291 |
| W | M | 6661 | 0,972 |  | W | M | 5871 | 0,830 |
| W | F | 3716 | 0,542 |  | W | F | 6311 | 0,893 |
| W | W | 3175 | 0,463 |  | W | W | 6159 | 0,871 |
| W | V | 6794 | 0,992 |  | W | V | 6451 | 0,912 |
| V | A | 6775 | 0,989 |  | V | A | 6713 | 0,949 |
| V | I | 6766 | 0,987 |  | V | I | 3608 | 0,510 |
| V | L | 6785 | 0,990 |  | V | L | 3699 | 0,523 |
| V | M | 6785 | 0,990 |  | V | M | 6402 | 0,905 |
| V | F | 5770 | 0,842 |  | V | F | 6733 | 0,952 |
| V | W | 5377 | 0,785 |  | V | W | 6583 | 0,931 |
| V | V | 6784 | 0,990 |  | V | V | 6783 | 0,959 |
|  | |  |  |  | |  |  |  |
|  | |  |  |  | |  |  |  |
| **Monomer C** | | | |  | **Monomer D** | | | |
| **Pos7** | **Pos30** | **Frequency** | **Normalized** |  | **Pos7** | **Pos30** | **Frequency** | **Normalized** |
| A | A | 6821 | 0,995 |  | A | A | 26 | 0,032 |
| A | F | 6585 | 0,961 |  | A | I | 4 | 0,005 |
| A | I | 6840 | 0,998 |  | A | L | 104 | 0,126 |
| A | L | 6841 | 0,998 |  | A | M | 54 | 0,066 |
| A | M | 6832 | 0,997 |  | A | V | 151 | 0,183 |
| A | V | 6849 | 0,999 |  | F | A | 3 | 0,004 |
| A | W | 6447 | 0,940 |  | F | I | 4 | 0,005 |
| F | A | 6788 | 0,990 |  | F | L | 22 | 0,027 |
| F | F | 4732 | 0,690 |  | F | M | 4 | 0,005 |
| F | I | 6536 | 0,953 |  | F | V | 18 | 0,022 |
| F | L | 6824 | 0,995 |  | I | A | 82 | 0,100 |
| F | M | 6819 | 0,995 |  | I | I | 8 | 0,010 |
| F | V | 6836 | 0,997 |  | I | L | 185 | 0,225 |
| F | W | 4867 | 0,710 |  | I | M | 136 | 0,165 |
| I | A | 6815 | 0,994 |  | I | V | 214 | 0,260 |
| I | F | 6042 | 0,881 |  | L | A | 270 | 0,328 |
| I | I | 6839 | 0,998 |  | L | F | 2 | 0,002 |
| I | L | 6835 | 0,997 |  | L | I | 85 | 0,103 |
| I | M | 6816 | 0,994 |  | L | L | 692 | 0,840 |
| I | V | 6839 | 0,998 |  | L | M | 447 | 0,542 |
| I | W | 6047 | 0,882 |  | **L** | **V** | **824** | **1,000** |
| L | A | 6843 | 0,998 |  | L | W | 32 | 0,039 |
| L | F | 6825 | 0,996 |  | M | A | 162 | 0,197 |
| L | I | 6842 | 0,998 |  | M | I | 30 | 0,036 |
| L | L | 6849 | 0,999 |  | M | L | 385 | 0,467 |
| L | M | 6829 | 0,996 |  | M | M | 364 | 0,442 |
| L | V | 6848 | 0,999 |  | M | V | 439 | 0,533 |
| L | W | 6815 | 0,994 |  | M | W | 1 | 0,001 |
| M | A | 6826 | 0,996 |  | V | A | 120 | 0,146 |
| M | F | 6809 | 0,993 |  | V | I | 25 | 0,030 |
| M | I | 6835 | 0,997 |  | V | L | 289 | 0,351 |
| **M** | **L** | **6855** | **1,000** |  | V | M | 206 | 0,250 |
| M | M | 6834 | 0,997 |  | V | V | 365 | 0,443 |
| M | V | 6849 | 0,999 |  | V | W | 6 | 0,007 |
| M | W | 6583 | 0,960 |  | W | A | 27 | 0,033 |
| V | A | 6784 | 0,990 |  | W | F | 3 | 0,004 |
| V | F | 6462 | 0,943 |  | W | I | 10 | 0,012 |
| V | I | 6801 | 0,992 |  | W | L | 8 | 0,010 |
| V | L | 6793 | 0,991 |  | W | M | 17 | 0,021 |
| V | M | 6794 | 0,991 |  | W | V | 140 | 0,170 |
| V | V | 6793 | 0,991 |  | |  |  |  |
| V | W | 6315 | 0,921 |  | |  |  |  |
| W | A | 6829 | 0,996 |  | |  |  |  |
| W | F | 6239 | 0,910 |  | |  |  |  |
| W | I | 6844 | 0,998 |  | |  |  |  |
| W | L | 6850 | 0,999 |  | |  |  |  |
| W | M | 6827 | 0,996 |  | |  |  |  |
| W | V | 6850 | 0,999 |  | |  |  |  |
| W | W | 5689 | 0,830 |  | |  |  |  |
